# Spatiotemporal assembly and functional composition of planktonic microeukaryotic communities along productivity gradients in a subtropical lake

**DOI:** 10.3389/fmicb.2024.1351772

**Published:** 2024-02-19

**Authors:** Songbao Zou, Qingping Lian, Meng Ni, Dan Zhou, Mei Liu, Xin Zhang, Guangmei Chen, Julin Yuan

**Affiliations:** ^1^Key Laboratory of Healthy Freshwater Aquaculture, Ministry of Agriculture and Rural Affairs, Huzhou, Zhejiang, China; ^2^Key Laboratory of Fish Health and Nutrition of Zhejiang Province, Huzhou, Zhejiang, China; ^3^Huzhou Key Laboratory of Aquatic Product Quality Improvement and Processing Technology, Huzhou, Zhejiang, China; ^4^Zhejiang Institute of Freshwater Fisheries, Huzhou, Zhejiang, China; ^5^Zhejiang Fenghe Fishery Co., Ltd., Lishui, Zhejiang, China

**Keywords:** plankton, microeukaryotic community, productivity, biogeography, functional traits, neutral community model

## Abstract

Microeukaryotes play crucial roles in the microbial loop of freshwater ecosystems, functioning both as primary producers and bacterivorous consumers. However, understanding the assembly of microeukaryotic communities and their functional composition in freshwater lake ecosystems across diverse environmental gradients remains limited. Here, we utilized amplicon sequencing of 18S rRNA gene and multivariate statistical analyses to examine the spatiotemporal and biogeographical patterns of microeukaryotes in water columns (at depths of 0.5, 5, and 10 m) within a subtropical lake in eastern China, covering a 40 km distance during spring and autumn of 2022. Our results revealed that complex and diverse microeukaryotic communities were dominated by Chlorophyta (mainly Chlorophyceae), Fungi, Alveolata, Stramenopiles, and Cryptophyta lineages. Species richness was higher in autumn than in spring, forming significant hump-shaped relationships with chlorophyll *a* concentration (Chl-*a*, an indicator of phytoplankton biomass). Microeukaryotic communities exhibited significant seasonality and distance-decay patterns. By contrast, the effect of vertical depth was negligible. Stochastic processes mainly influenced the assembly of microeukaryotic communities, explaining 63, 67, and 55% of community variation for spring, autumn, and both seasons combined, respectively. Trait-based functional analysis revealed the prevalence of heterotrophic and phototrophic microeukaryotic plankton with a trade-off along N:P ratio, Chl-*a*, and dissolved oxygen (DO) gradients. Similarly, the mixotrophic proportions were significantly and positively correlated with Chl-*a* and DO concentrations. Overall, our findings may provide useful insights into the assembly patterns of microeukaryotes in lake ecosystem and how their functions respond to environmental changes.

## Introduction

Microeukaryotic plankton, encompassing a broad range of unicellular eukaryotes like algae, protozoa, and fungi, are vital components within microbial food webs (Azam and Malfatti, 2007). More importantly, the microplankton community contributes significantly to primary productivity and nutrient recycling in freshwater environments (Liu et al., 2013; Gad et al., 2020). The functions and community dynamics of planktonic microeukaryotes exert a profound influence on water quality (Yu et al., 2014; Gad et al., 2022). Adverse events like algal blooms and toxin production, induced by community variation, adversely affect sources of drinking water (Zhang et al., 2018; Treuer et al., 2021). Therefore, planktonic microeukaryote communities are commonly employed as biomonitors for assessing aquatic ecosystem health and water quality (Foissner and Berger, 1996). Over the past decades, much effort has been made to unveil how the freshwater microeukaryotic communities are distributed and assembled (Chen et al., 2019; Isabwe et al., 2019; Abdullah Al et al., 2022). The biogeography of microeukaryotic planktons can be shaped by abiotic factors (e.g., nutrients, temperature, light, pH, depth, spatial factors) and biotic factors (including top-down predation, competition, mutualism, and trade-offs) (Gong et al., 2015; Wang et al., 2015; Chen et al., 2019; Wang et al., 2020). Nevertheless, discerning the relative significance of these factors in determining community structure and understanding the variability of those driving factors across spatial, vertical, and seasonal scales, remains fundamental issues in freshwater ecology. Furthermore, understanding how freshwater microeukaryotic community dynamics evolve is critical for ecosystem functionality prediction and management in the context of global change.

Pigmented microeukaryotes are key constituents of the phytoplankton realm in aquatic ecosystems (Xu et al., 2018). The concentration of chlorophyll *a* (Chl-*a*), a reliable surrogate for photosynthetic potential and primary productivity, often demonstrates a hump-shaped relationship with the diversity of phytoplankton in aquatic environments (Vallina et al., 2014). The productivity-diversity relationships depend on scale of investigations (Chase and Leibold, 2002), the type of water body (Borics et al., 2014), and are susceptible to the influence of environmental change (Righetti et al., 2019). Studies have documented that phytoplankton release a substantial proportion of photosynthate, ranging from 0 to 80% of that fixed via photosynthesis, as dissolved organic matter (DOM) into the surrounding waters (Hulatt and Thomas, 2010). The DOM is directly incorporated into the microbial loop via heterotrophic bacterial consumption, subsequently enhancing the abundance of bacterivorous (i.e., heterotrophic/mixotrophic) microeukaryotes. In a recent investigation, an uncommon U-shaped pattern was discovered between microeukaryotes and Chl-*a* in eutrophic coastal oceans (Wang et al., 2020). Nevertheless, within freshwater ecosystems, there is a lack of comprehensive exploration into the variations in microeukaryotic community structure linked to productivity and the primary environmental factors responsible for driving these changes.

Identifying and quantifying the ecological processes that regulate the assembly of microbial communities in aquatic ecosystems stands as a central focus in microbial ecology (Logares et al., 2013; Nemergut et al., 2013). Addressing this critical issue is essential for advancing our comprehension of diversity, functioning, and successional dynamics of microorganisms (Liu et al., 2019). Classical niche-based theory proposes that deterministic processes, involving both biotic interactions and abiotic factors, jointly influence community structure (Chesson, 2000; Fargione et al., 2003). However, in neutral theory of biodiversity, species are viewed as functionally equivalent, with their abundances primarily determined by stochastic processes such as birth, death, speciation, extinction, and dispersal (Hubbell, 2001; Chave, 2004). Deterministic processes mainly governed the assembly of microeukaryotic communities in estuarine, coastal, and marine ecosystems (Wu et al., 2018; Zhu et al., 2021; Wu et al., 2023). However, in freshwater environments such as rivers and lakes, as well as surface ocean, microeukaryotic structure exhibited a pronounced influence of stochastic processes (Chen et al., 2019; Logares et al., 2020; Ren et al., 2022; Han et al., 2023). Frequently, microeukaryotic communities seem to be regulated by a combination of both deterministic and stochastic processes (Gad et al., 2020; Hou et al., 2020; Mo et al., 2021). Nonetheless, a comprehensive understanding of microeukaryotic geographical patterns and assembly mechanisms in freshwater ecosystems across spatial, temporal, vertical water column, and environmental gradients is still lacking.

The Qianxia Lake (E119.9 N28.1), located in Qingtian County, Zhejiang Province, China, is a fjord-type lake with a long and narrow watershed covering approximately 3,330 km^2^. It spans 105 km in length and averages 31.7 km in width (Yao et al., 2022). The lake is a crucial source of drinking water for numerous towns and sustains agriculture, power generation, and tourism, with minimal industrial pollution. Thus, the Qianxia Lake watershed offers an ideal setting to explore the relative importance of environmental and spatial factors on community composition. This knowledge is crucial for integrating planktonic microeukaryotic components into ecosystem and biogeographical models to improve predictive accuracy. Furthermore, given the dispersal capacity of microplankton communities in lotic habitats, it renders them well-suited for investigating the impact of dispersal on community variation and the influence of seasonal changes on dispersal.

In this investigation, we conducted a comparative analysis of microeukaryotic plankton communities in the Qianxia lake, employing amplicon sequencing of 18S rRNA genes. Our data included 60 water samples collected across two seasons and various water depths along the Qianxia Lake (Supplementary Figure S1), with the following objectives: (1) Unraveling the spatiotemporal distribution of microeukaryotic communities and deciphering the underlying mechanism governing their assembly; (2) Investigating how the microeukaryotic diversity and community structure respond to productivity gradients; and (3) Assessing the impact of environmental factors on variation in functional composition of microeukaryotic communities.

## Materials and methods

### Sampling and determination of environmental parameters

Sampling of water was carried out at 8 sites in spring and 12 sites in autumn across the Qianxia Lake (119°65′–120°04′ E, 27°99′–28°13′ N) in April and September 2022 (Supplementary Figure S1). A total of 60 water samples were collected at depths of 0.5, 5, and 10 m in the epilimnion zone of water column. Due to the lake’s depth ranging from 10.5 to 79.4 m, deep samples were collected away from the lakebed to prevent disturbance to sediment (Supplementary Table S1). The water collection utilized 5 L Niskin bottles, pre-filtered through a 200 μm mesh to eliminate larger plankton and debris. Subsequently, water (~500 mL) with microeukaryotes (size <200 μm) was vacuum-filtered onto a 0.22 μm filter (Supor 200, PALL, Michigan, United States). All membranes were placed into 2 mL cryotubes and preserved in liquid nitrogen for DNA extraction.

The *in situ* lake water’s physicochemical parameters (temperature, pH, and dissolved oxygen) were determined through a water quality analyzer (HQ40d, Hach company, United States). The Chl-*a* concentration was measured employing an electronic sensor (TriBox mini, Germany). Determination of nitrate (NO_3_-N), nitrite (NO_2_-N), ammonium (NH_4_^+^-N), total nitrogen (TN), and soluble reactive phosphate (PO_4_^3−^) in all sub-samples via an auto-analyzer (Seal, Germany). Total phosphorus (TP), COD_Mn_, and total organic carbon (TOC) were determined through spectrophotometry following standard methods. A total of 12 environmental parameters were determined in this study (Supplementary Table S1).

### DNA preparation and high-throughput sequencing

We extracted DNA using the PowerSoil^®^ DNA Isolation Kit (MoBio, Carlsbad, CA), following the manufacturer’s protocol. The assessment of DNA concentration and quality was performed with an NC2000 spectrophotometer (Thermo Scientific, United States). PCR amplification targeted the V4 region of microeukaryotic 18S rRNA gene, employing the specific primers 565F (5′-CCAGCASCYGCGGTAATTCC-3′) and 948R (5′-ACTTTCGTTCTTGATYRA-3′) (Stoeck et al., 2010). PCR reactions followed this procedure: 98°C for 2 min, followed by 25 cycles of 98°C for 15 s, 55°C for 30 s, and 72°C for 30 s; and a final extension at 72°C for 5 min. Paired-end (2 × 250 bp) sequencing was conducted using the Illumina NovaSeq platform (Illumina, United States) at Personalbio company (Shanghai, China).

### Bioinformatics

The raw sequence data were initially demultiplexed, and primers were trimmed with Cutadapt v1.18 (Martin, 2011), followed by processing through the DADA2 pipeline (Callahan et al., 2016) in R v4.0.0 (R Core Team, 2018). The parameter settings followed our prior study (Zou S. et al., 2021). Briefly, sequencing reads were quality filtering with the “filterAndTrim” function. The dereplication process generated unique sequences, implemented with “derepFastq” function; Error models were trained with the “learnErrors” function. ASVs were inferred using the core function “dada.” Paired reads were merged using the “mergePairs” function, and bimeras were eliminated with the “removeBimeraDenovo” function. Taxonomic classification was performed using the *DECIPHER* package’s “IdTaxa” function against the PR^2^ v2.0 database (Wright, 2016). ASVs that were unassigned or appeared as singletons, as well as reads from Metazoa, Ulvophyceae, Streptophyta, and Rhodophyta, were excluded from subsequent analysis.

Prior to computing alpha diversity metrics, all sample were rarefied to the lowest sequencing depth of 18,300 reads (Supplementary Table S3), which minimize sequencing depth bias and enable comparable diversity assessments. To analyze beta diversity analysis, we normalized the ASV table using *edgeR* package (Robinson et al., 2010), following the recommendation of McMurdie and Holmes (2014). Bray-Curtis dissimilarities distances were computed and then visualized in NMDS plot using the “metaMDS” function of the *vegan* package (Oksanen et al., 2013).

### The relative contribution of environmental and spatial factors in structuring microeukaryotic communities

We assessed the spatial distribution of microeukaryotic communities by assessing the community turnover rate (*z* value) over spatial distances, as proposed by Ranjard et al. (2013): log_10_(χd) = (−2*z*) * log_10_(d) + b. The pairwise Sørensen similarity (χd) was computed using the “dsvdis” function from the *labdsv* package (Roberts, 2019). The distance (d) between sampling sites (in meters) was determined using the function “distm” of *geosphere* package (Hijmans et al., 2017). The “b” represents the intercept of the linear model.

We further employed variation partitioning analysis (VPA) to assess the contribution of environmental and spatial factors in microeukaryotic assembly (Borcard et al., 1992). All environmental factors (except pH) underwent square root transformation for normality and homoscedasticity. For spatial factors, we initially converted the longitude and latitude to the Cartesian coordinates using the function “geoXY” in the *SoDA* package (Chambers, 2008). Subsequently, an Euclidean distance matrix was generated from these Cartesian coordinates using the “dist” function. The “PCNM” function (permutations = 1,000, *vegan* package) was then applied to this matrix (Borcard and Legendre, 2002).

Redundancy analysis (RDA) was chosen to explore the relationship between microeukaryotic communities and environmental/spatial factors based on the longest gradient length of detrended correspondence analysis (DCA). Preceding RDA, the following steps were executed: (1) Hellinger transformation of microeukaryotic data; (2) Variance inflation factor (VIF) was calculated using the “vif.cca” function (*vegan* package) and variables with VIF > 10 were discarded to address the multicollinearity effects; and (3) Identification of significant (*p* < 0.05) explanatory factors through forward selection using the “ordiR2step” function (Blanchet et al., 2008). The relative effects of environmental and spatial factors on community variation were determined with VPA using the “varpart” function of package *vegan* in R, and the significance of each fraction was determined using the permutation test with the “anova.cca” function.

### Functional analysis based on taxonomic specifically traits approaches

To elucidate the functional composition of microeukaryotes and their responses to the environmental gradients, we utilized a trait-based approach, as previously described (Genitsaris et al., 2015; Adl et al., 2019; Ramond et al., 2019; Wang et al., 2020; Pan et al., 2022). In brief, the taxonomic assignments of microeukaryotic members, as annotated by the PR^2^ database, were converted into ecological traits, while retaining their read proportions within a given community. In this study, the trophic traits were simplified and categorized into phototrophy, heterotrophy, and mixotrophy (see Supplementary Table S4). The ASV richness and abundance for these three functional groups were determined by summing the taxonomic groups within each corresponding functional category.

### Estimation of importance of environmental variables using random forest model

We utilized random forest analysis to evaluate the impact of environmental factors on the functional composition of microeukaryotes, employing the *rfPermute* package with 999 permutations (Archer, 2019). The analysis of percentage increases in mean squared error (MSE%) of predictors determined the importance of the variables. Variables with higher MSE% values were implied as more influential. Model significance and cross-validated *R*^2^ were assessed using the *A3* package in R (Fortmann-Roe, 2015).

### Fitting the neutral community model for microeukaryotes

We evaluated the influence of stochastic processes on the microeukaryotic community assembly using the neutral community model (NCM), as described by Sloan et al. (2006). The NCM employs *R*^2^ for the fit to the neutral model and *Nm* values as the product of metacommunity size (*N*) and immigration rate (*m*). Additionally, we compared the richness and abundance of microeukaryotes in neutral and non-neutral (above and below) partitions to evaluate the deviations from the NCM predictions. All computations and visualizations were performed using R.

### Statistical analyses

Two-tailed student’s *t*-tests and one-way ANOVA with least significant difference (LSD) *post-hoc* were utilized to assess the variations in physicochemical variables, alpha diversity metrics, relative proportions of major taxonomic taxa between seasons, among depths, and across Chl-*a* levels. Permutational multivariate analysis of variance (PERMANOVA, 999 permutations) was employed to examine differences in community structure among groups based on Bray-Curtis distances (Anderson, 2001). All geographic measurements were calculated and visualized with ArcGIS v.10.8 (Redlands, California, United States). The relationships between environmental or spatial factors and taxonomic/functional diversity were assessed through Pearson or Spearman’s rank correlations analysis using SPSS v.13.0 (IBM, United States).

## Results

### Seasonal and vertical variations in abiotic variables

The physicochemical variables determined are presented in Supplementary Table S1. Of the 12 environmental parameters, more than half demonstrated a distinct seasonality. The water temperature ranged from 19.1 to 27.3°C in spring and from 26.4 to 29.9°C in autumn (*p* < 0.001). Significant differences were observed in Chl-*a* concentration, which was higher in spring (mean ± SE, 5.901 ± 0.502 μg/L) than in autumn (1.895 ± 0.203 μg/L; *p* < 0.001). Similarly, levels of DO (9.598 ± 0.138 vs. 8.069 ± 0.078 mg/L), NO_2_-N (0.004 ± 0 vs. 0.003 ± 0 mg/L; *p* < 0.001), pH (9.435 ± 0.133 vs. 8.694 ± 0.079; *p* < 0.001), and TOC (70.208 ± 2.576 vs. 58.833 ± 0.746 mg/L; *p* < 0.001) showed spring-autumn distinctions. However, concentrations of TP (0.421 ± 0.011 vs. 1.350 ± 0.027 mg/L; *p* < 0.001) and PO_4_-P (0.124 ± 0.009 vs. 0.153 ± 0.010 mg/L; *p* < 0.001) were relatively lower in spring due to increased nutrient uptake by high abundance of phytoplankton. In terms of depths, environmental factors were relatively stable, although the concentrations of DO, pH, and temperature significantly decreased with depth, while TN tended to increase with depth (Supplementary Table S2).

### Differences in alpha diversity and community composition of planktonic microeukaryotes across seasons and depths

**Figure 1:**
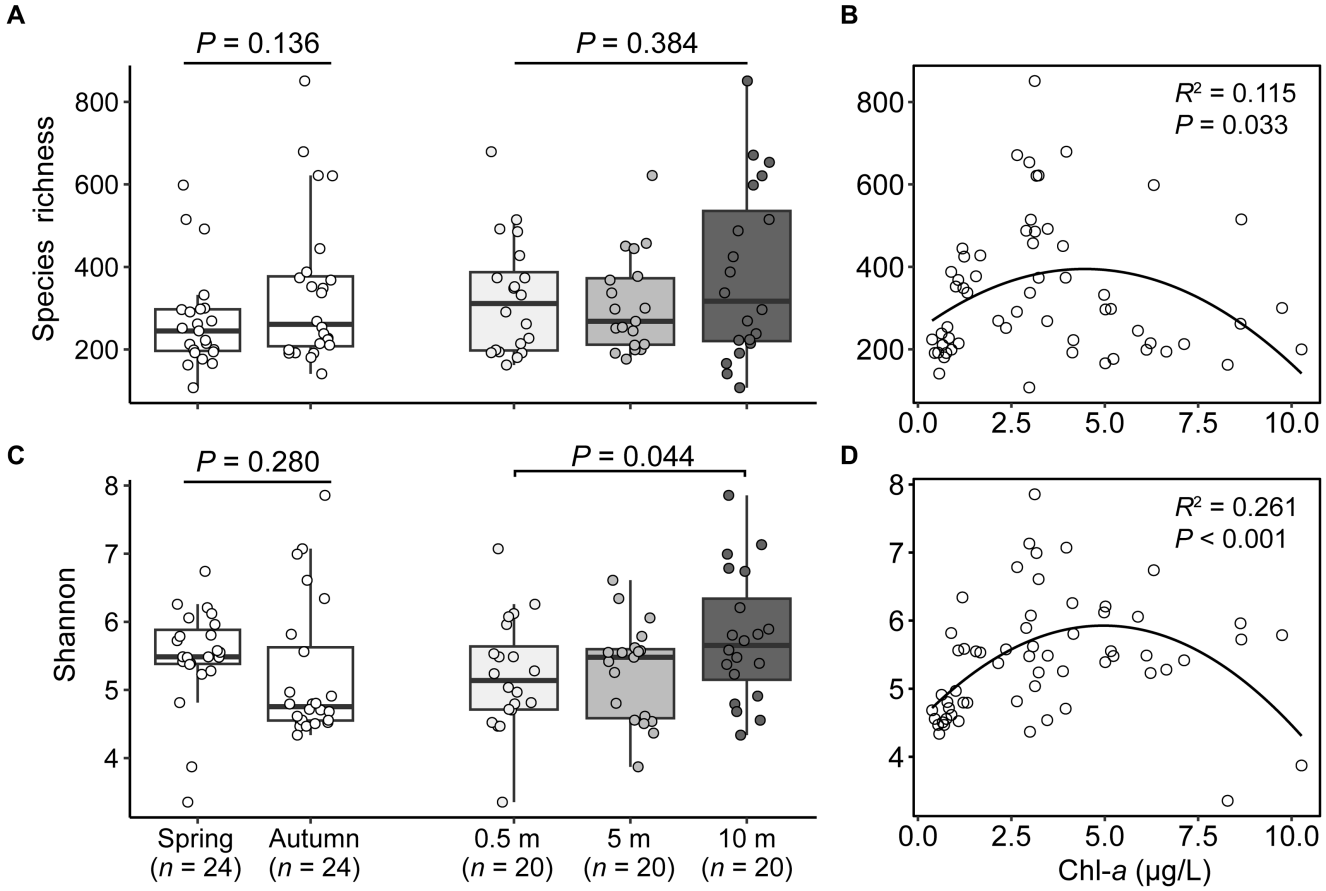
Seasonal and vertical variations in species richness **(A)** and Shannon diversity **(C)** of microeukaryotic plankton, and both fitted significant hump-shaped relationships with concentrations of chlorophyll *a* **(B,D)**.

A total of 6,383 ASVs were generated from 6,520,323 high-quality sequences for 60 samples, among which 2,758 and 4,059 ASVs were detected in spring and autumn, respectively (Supplementary Table S3). Of the rarefied dataset, the alpha-diversity metrics of microeukaryotes exhibited considerable variability among samples, with ASV richness ranging from 107.3 to 850.9 and Shannon indices between 3.4 to 7.9 (Supplementary Table S3). In contrasting the two seasons, autumn had higher species richness (338.1) compared to spring (269.4), while Shannon indices lower in autumn than in spring. Among the three depths, the alpha-diversity estimators usually increased with the depths (Figures 1A,C). The species richness and Shannon both formed significant hump-shaped relationships with concentrations of Chl-*a* (*R*^2^ ≥ 0.115, *p* ≤ 0.033), and the species richness appears to peak at the concentrations of Chl-*a* of 4.0 μgL^−1^ (Figures 1B,D).

**Figure 2:**
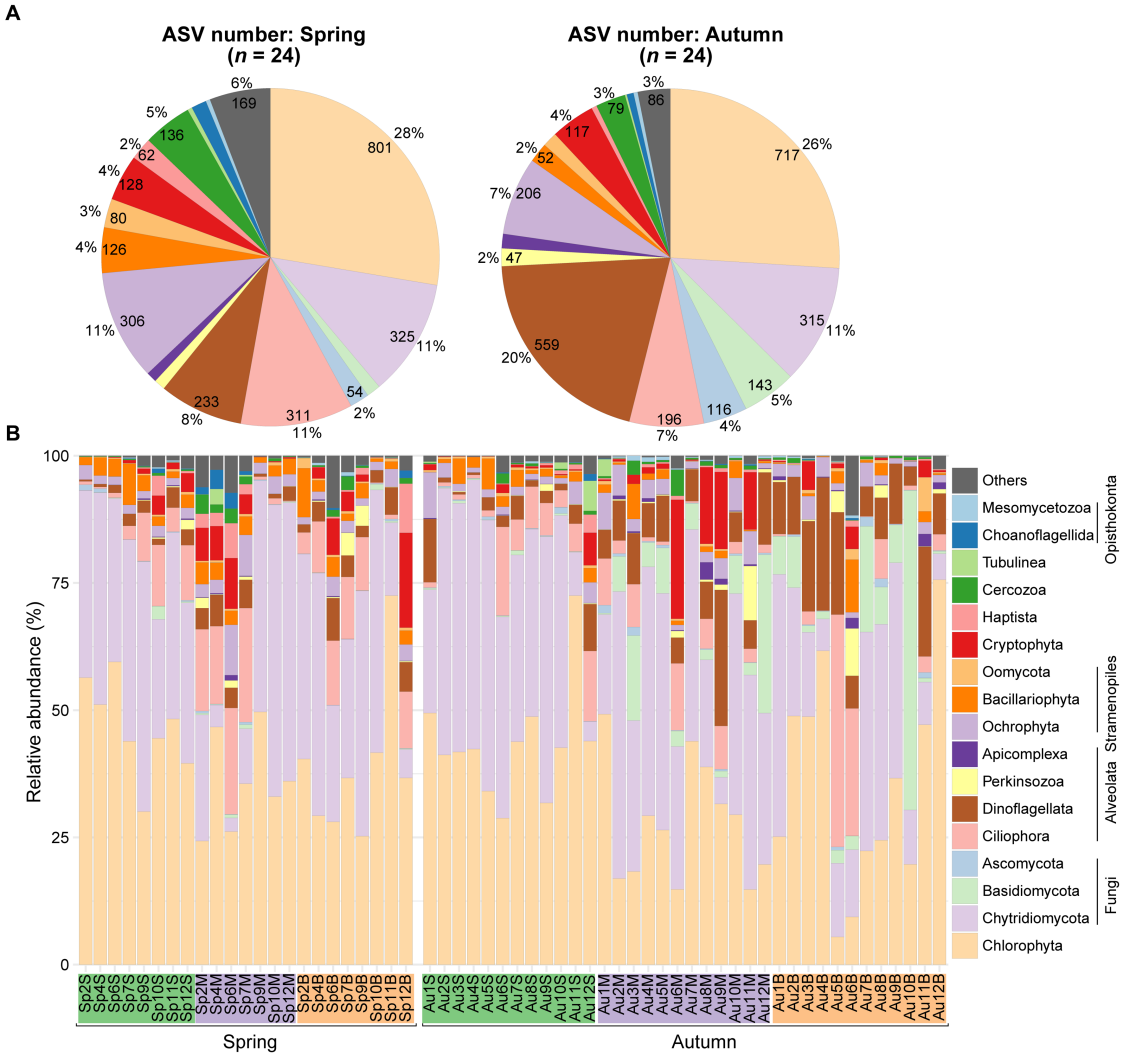
Differences in diversity and composition of planktonic microeukaryotes community. **(A)** Pie plots depict ASV numbers for major taxa during spring and autumn, respectively. **(B)** Bar plot illustrates relative proportions of major taxa across the samples.

The ASV number were mainly affiliated with phylum Chlorophyta (on average 27%), followed by Dinoflagellata (14%), Chytridiomycota (11%), Ciliophora (9%), Ochrophyta (9%), Cercozoa (4%), Cryptophyta (4%). Seasonal variations were noted, with higher occurrences of ASVs in the spring compared to the autumn for Ciliophora (11% vs. 7%), Ochrophyta (11% vs. 7%), and Cercozoa (5% vs. 3%), but the opposite for Dinoflagellata (8% vs. 20%) (Figure 2A).

The reads of microeukaryotes exhibited dominance by Chlorophyta, accounting for a relative abundance of 37.5%, followed by Fungi at 36.3%, mainly composed of Chytridiomycota and Basidiomycota. Other notable phyla included Alveolata (14.2%), Stramenopiles (5.6%), and Cryptophyta (2.9%). Within Alveolata, Ciliophora comprised 6.6% and Dinoflagellata constituted 6.4%. Stramenopiles were predominantly represented by Ochrophyta (2.9%) and Bacillariophyta (2.3%). Reads proportions of other taxa (e.g., Haptista, Cercozoa, Amoebozoa, and Opisthokonta) averaged less than 1% (Figure 2B).

Additionally, the ASV richness in Dinoflagellata, Basidiomycota, Oomycota, and Perkinsozoa exhibited significant hump-shaped relationships with Chl-*a*, except for Chytridiomycota and Bacillariophyta, which formed negative relationships with Chl-*a* (0.083 ≤ *R*^2^ ≤ 0.357, *p* ≤ 0.044; Supplementary Figure S2).

### Changes in taxonomic composition between seasons, among depths, and along productivity gradients

**Figure 3:**
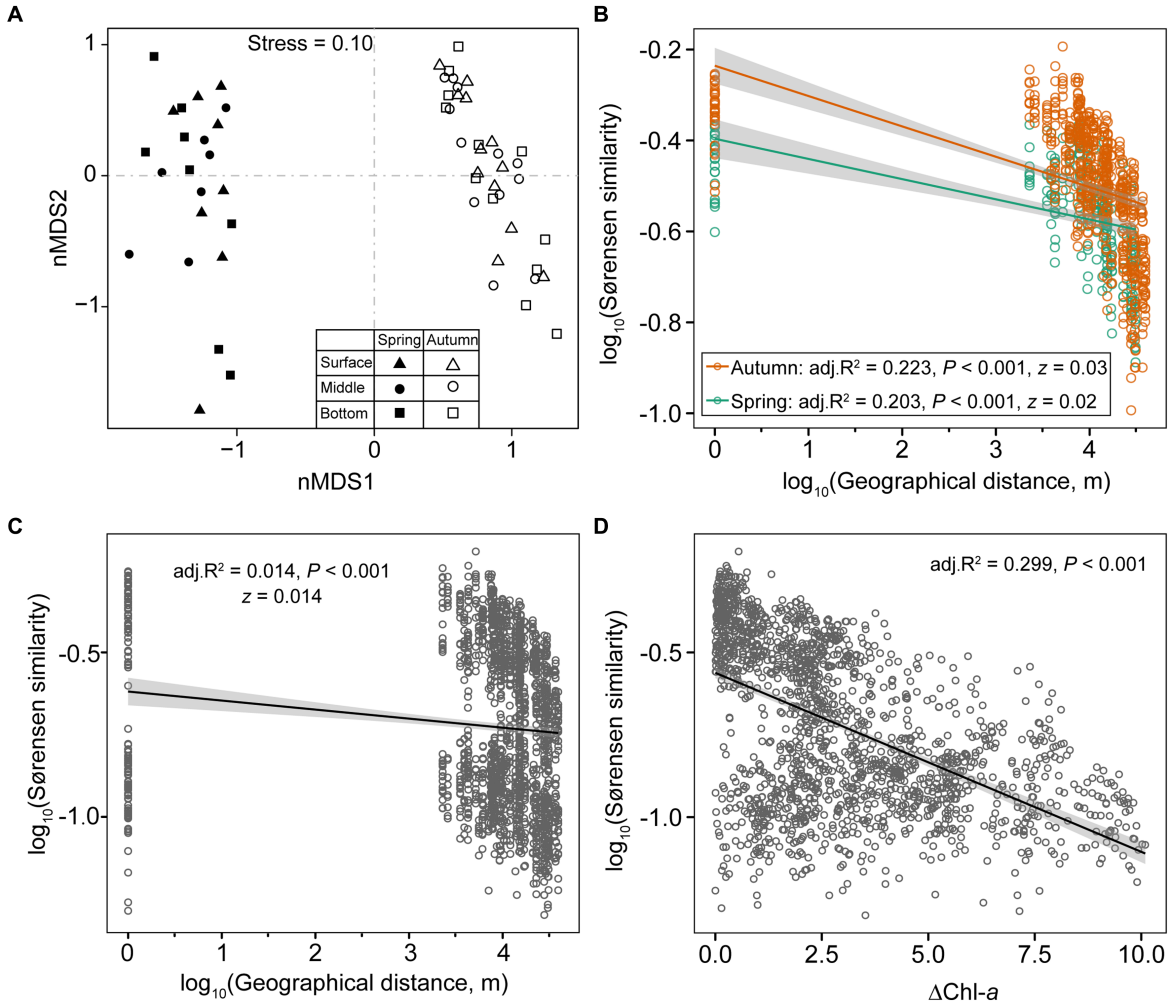
nMDS using Bray–Curtis dissimilarity illustrates the seasonal variations in planktonic microeukaryotic communities **(A)**. Scatter plots depict distance-decay patterns between Sørensen similarity of communities and pair-wise geographic distance in spring, autumn, and both seasons **(B,C)**. Sørensen similarities of communities also show a negative correlation with the gradients of productivity **(D)**.

The NMDS plot and PERMANOVA analysis indicated a distinct separation in microeukaryotic community compositions between spring and autumn (*R*^2^ = 0.302, *p* = 0.001). However, samples from different depths did not exhibit clear differentiation (*R*^2^ = 0.025, *p* = 0.79; Table 1; Figure 3A). The log-transformed Sørensen similarity exhibited significant distance-decay patterns in microeukaryotic community for both seasons (adj. *R*^2^ ≥ 0.203; *p* < 0.01, Figure 3B). The turnover rate (*z* value) was slightly higher in autumn than in spring (0.03 vs. 0.02, Figure 3C). Similarly, microeukaryotic community similarities between any two sampling sites significantly decreased with pairwise differences in Chl-*a* concentrations (adj. *R*^2^ ≥ 0.299; *p* < 0.001 Figure 3D), highlighting the crucial role of Chl-*a* in structuring microeukaryotic assembly. Additionally, similarity of planktonic microeukaryotes, spanning taxonomic ranks from species to phylum, also demonstrated pronounced distance-decay patterns (*p* < 0.001; Supplementary Figure S3).

**Table 1:** ADONIS analysis to assessed microeukaryotic communities variations using Bray-Curtis and Sørensen distances across seasons, and among depths and levels of chlorophyll-*a*.

Grouping by	Bray-Curtis		Sørensen	
	*R* ^2^	*p*	*R* ^2^	*p*
Season	0.302	**0.001**	0.191	**0.001**
Depth (Global test)	0.025	0.79	0.028	0.834
0.5 m vs. 5 m	0.018	0.962	0.018	0.959
0.5 m vs. 10 m	0.023	0.563	0.023	0.533
5 m vs. 10 m	0.023	0.598	0.023	0.594
Chl-*a* (Global test)	0.296	**0.001**	0.206	**0.001**
0–2 vs. 2–5	0.182	**0.001**	0.133	**0.001**
0–2 vs. 5–11	0.381	**0.001**	0.249	**0.001**
2–5 vs. 5–11	0.176	**0.001**	0.119	**0.001**

Significant differences in pair-wise comparison were bold.

**Figure 4:**
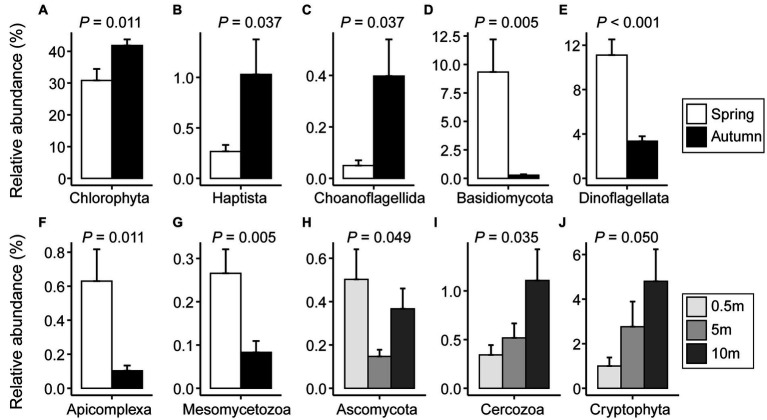
The relative proportions of microeukaryotic taxa with significant differences between spring and autumn **(A–G)** or among vertical depths of water column **(H–J)**. Significance was assessed through Student’s *t*-test or one-way ANOVA test (*p* < 0.05). The error bars denote standard errors.

Comparing the proportions of major microeukaryotic taxa in reads between seasons revealed that Chlorophyta (41.8 vs. 30.8%), Haptista (1.03 vs. 0.27%), and Choanoflagellida (0.40 vs. 0.05%) were significantly more prevalent in autumn than in spring. In contrast, Basidiomycota (0.28 vs. 9.34%), Dinoflagellata (3.35 vs. 11.1%), Apicomplexa (0.10 vs. 0.63%), and Mesomycetozoa (0.08 vs. 0.27%) exhibited an opposite trend (Figures 4A–G; Supplementary Table S5). Regarding the depth, the variations were mainly attributed to Ascomycota, Cercozoa, and Cryptophyta (Figures 4H–J; Supplementary Table S5).

**Figure 5:**
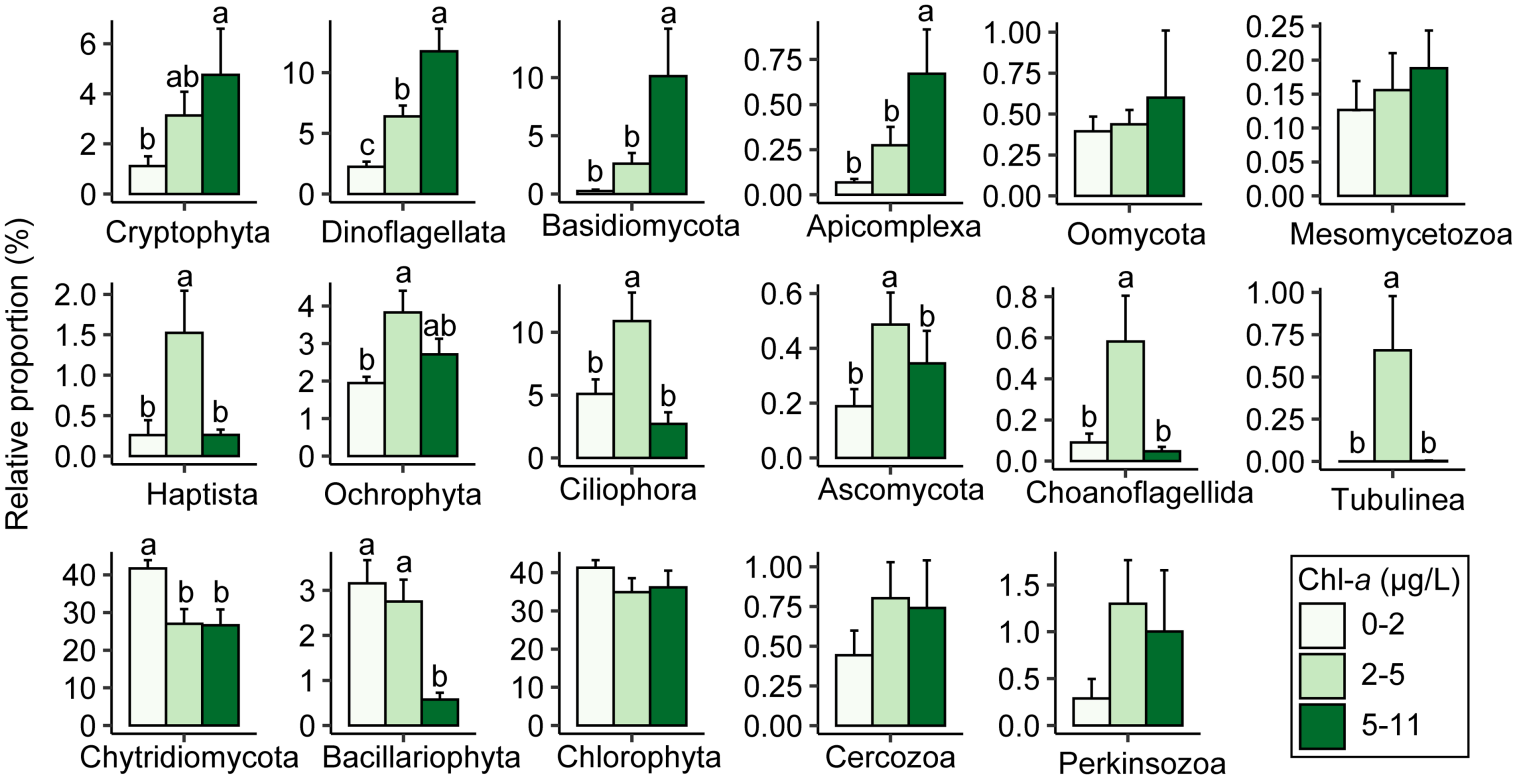
Comparisons in relative proportions of reads assigned to each taxa among levels of chlorophyll-*a* by one-way ANOVA. Distinct lowercase letters signify significant variations (*p* < 0.05).

To explore the impact of the productivity gradients on microeukaryotic community structure, we statistically compared the relative proportions of major taxa at three Chl-*a* levels (0–2 μg L^−1^ for low level; 2–5 μg L^−1^ for intermediate level; and 5–11 μg L^−1^ for high level; Figure 5; Supplementary Table S5). Proportions of Cryptophyta, Dinoflagellata, Basidiomycota, and Apicomplexa increased with levels of Chl-*a*. Under intermediate levels, six taxonomic groups (Haptista, Ochrophyta, Ciliophora, Ascomycota, Choanoflagellida, and Tubulinea) had the highest relative abundance. However, Chytridiomycota and Bacillariophyta were more abundant in low concentrations of Chl-*a* (Figure 5).

### Variations in functional structure of microeukaryotic communities

**Figure 6:**
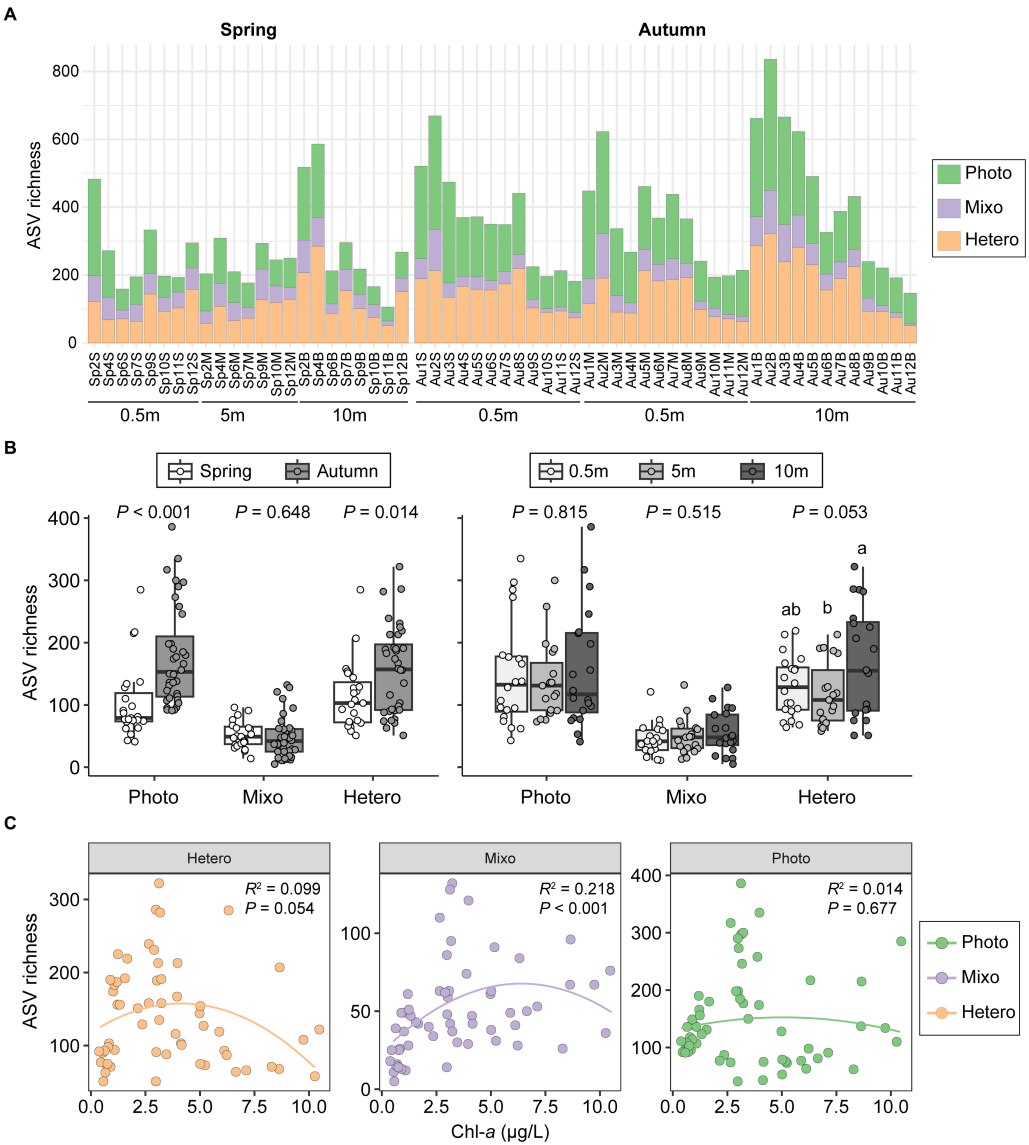
Differences in community richness among samples for phototrophic, mixotrophic, and heterotrophic groups **(A)**. Comparisons between two seasons and among vertical depths **(B)**. The mixotrophs formed a significant humped relationship with chlorophyll-*a* concentrations, contrasting with the heterotrophs and phototrophs **(C)**. *T*-test and ANOVA were employed to assess differences in richness between seasons and among depths. Photo, phototrophs; Mixo, mixotrophs; Hetero, heterotrophs.

The ASV richness of phototrophs varied greatly across all samples (51–322) and was comparable to that of heterotrophs (41–386). Phototrophs and heterotrophs were both nearly three times more abundant than mixotrophs (Figure 6A). Seasonally, phototrophs and heterotrophs showed similar patterns, with significantly higher abundances in autumn than in spring (*p* ≤ 0.04). Conversely, mixotrophs richness was slightly higher in spring, although this difference did not reach statistical significance (*p* = 0.648; Figure 6B). Regarding depth-related comparisons, a notable difference in richness was evident solely for heterotrophs between bottom layer and middle layer (55.5 vs. 49.4; *p* < 0.05; Figure 6B). The correlations between the richness of three functional groups and Chl-*a* concentrations were weak and lacked statistically support, except for mixotrophs (*R*^2^ = 0.218, *p* < 0.001; Figure 6C).

**Figure 7:**
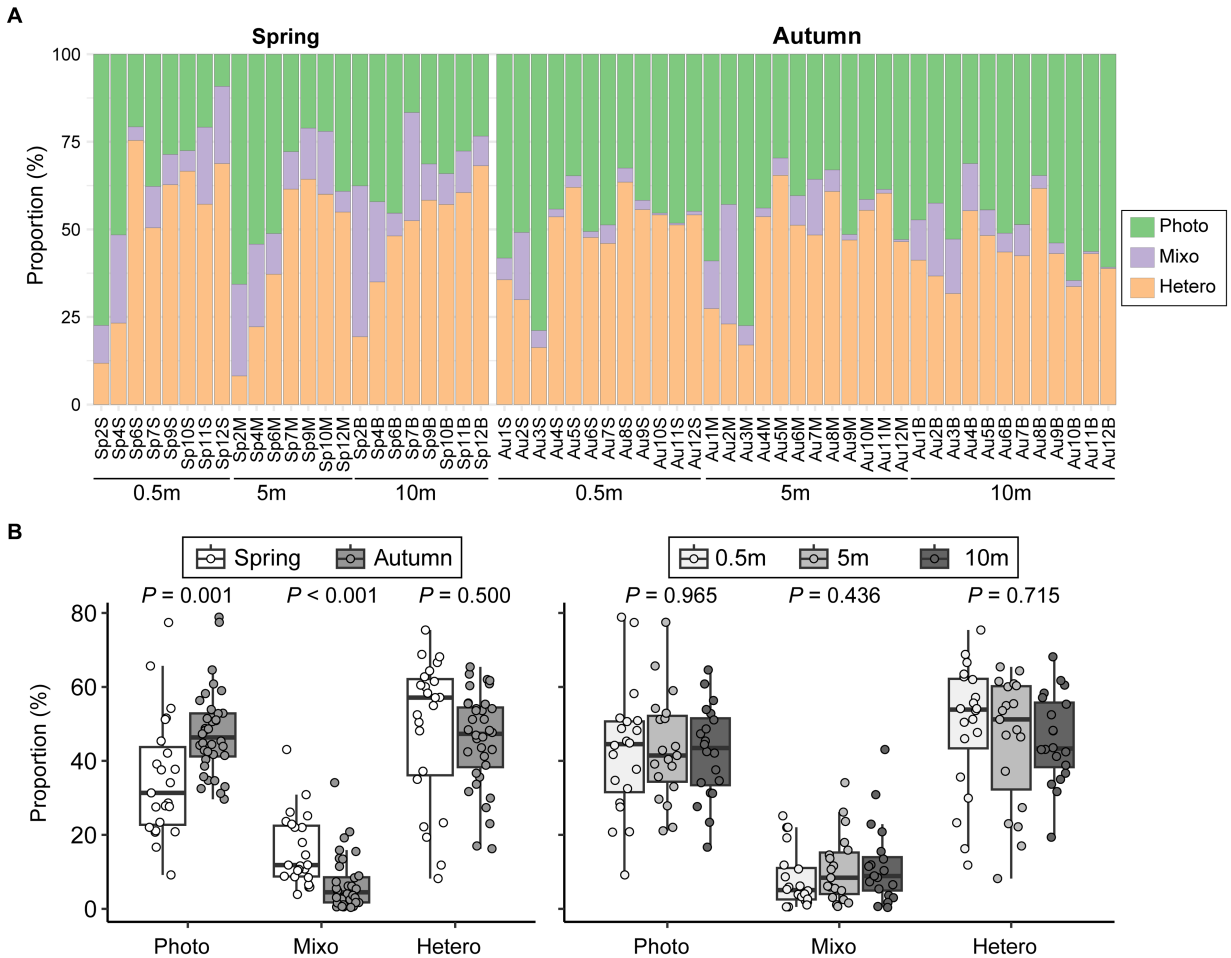
Variations in functional composition of microeukaryotic communities among samples **(A)** and comparisons between seasons and among vertical depths **(B)**. Photo, phototrophs; Mixo, mixotrophs; Hetero, heterotrophs.

Seasonal variations significantly influenced the relative abundances of the three functional groups of microeukaryotes (Figure 7A). Heterotrophs and phototrophs dominated the microeukaryotic communities among all samples, accounting for an average percentage of 46.9 and 42.8%, respectively, while mixotrophs consistently had a low contribution (10.2%). Higher abundances of mixotrophs and heterotrophs were detected in spring, while phototrophs became more abundant in autumn (*p* = 0.001). However, vertical comparisons did not reveal any significant differences in reads proportions of functional groups (*p* > 0.436; Figure 7B).

**Figure 8:**
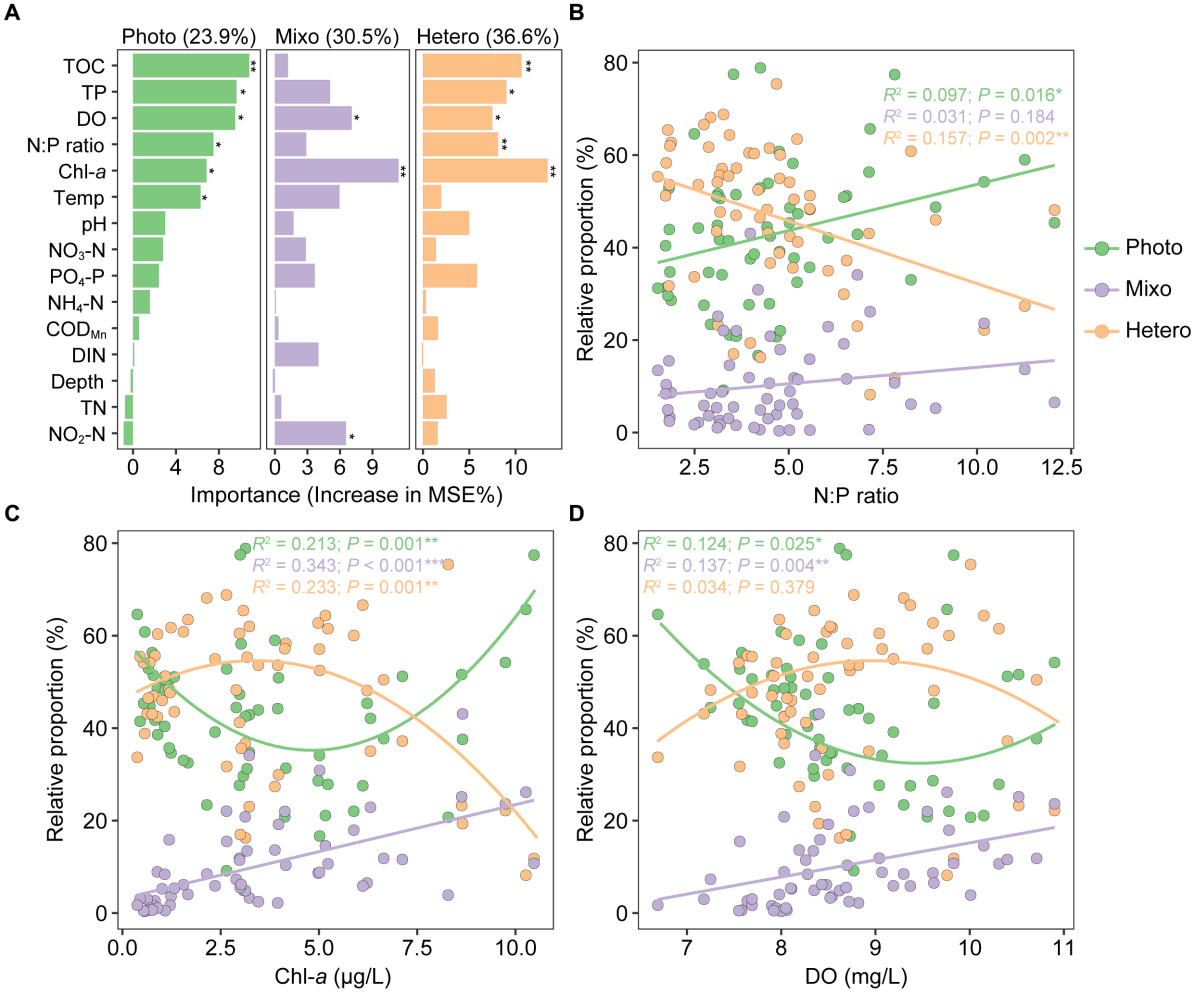
Drivers of planktonic microeukaryotic functional composition variation in Qianxia Lake. Random forest algorithm was used to determine the importance of environmental factors in explaining the variations of functional groups **(A)**. The relative proportions of phototrophs, mixotrophs, and heterotrophs covaried well with several environmental gradients, including N:P ratio **(B)**, Chl-*a* **(C)**, and dissolved oxygen **(D)**. Statistical significance is indicated as follows: *p < 0.05, **p < 0.01, and ***p < 0.001.

Random forest analysis was utilized to assess the significance of environmental factors in shaping functional composition. The results revealed the highest contribution to heterotrophs (36.6%), followed by mixotrophs (30.5%), and phototrophs (23.9%). These variations were predominantly driven by a combination of environmental factors, including Chl-*a*, N:P ratio, DO, TP, TOC, temperature, and NO_2_-N (*p* < 0.05, Figure 8A). Relationships between the relative abundances of functional groups and key environmental factors exhibited distinct trends: the proportion of phototrophs showed a significant and positive correlation with N:P ratio (*R*^2^ = 0.097, *p* = 0.016), whereas heterotrophs exhibited a significant and negative correlation with N:P ratio (*R*^2^ = 0.157, *p* = 0.002). Nevertheless, the correlation between mixotrophs and N:P ratio was weak and non-significant (*R*^2^ = 0.031, *p* = 0.184; Figure 8B). Across the gradients of Chl-*a*, phototrophs, heterotrophs, and mixotrophs exhibited hump-shaped, U-shaped, and positive linear patterns, respectively. Similarly, these relationships were in parallel with dissolved oxygen gradients but with lower correlation coefficients, and only significant for phototrophs and mixotrophs (Figures 8C,D).

### Relative contribution of environmental drivers to variations in microeukaryotic communities and underlying assembly mechanisms

**Figure 9:**
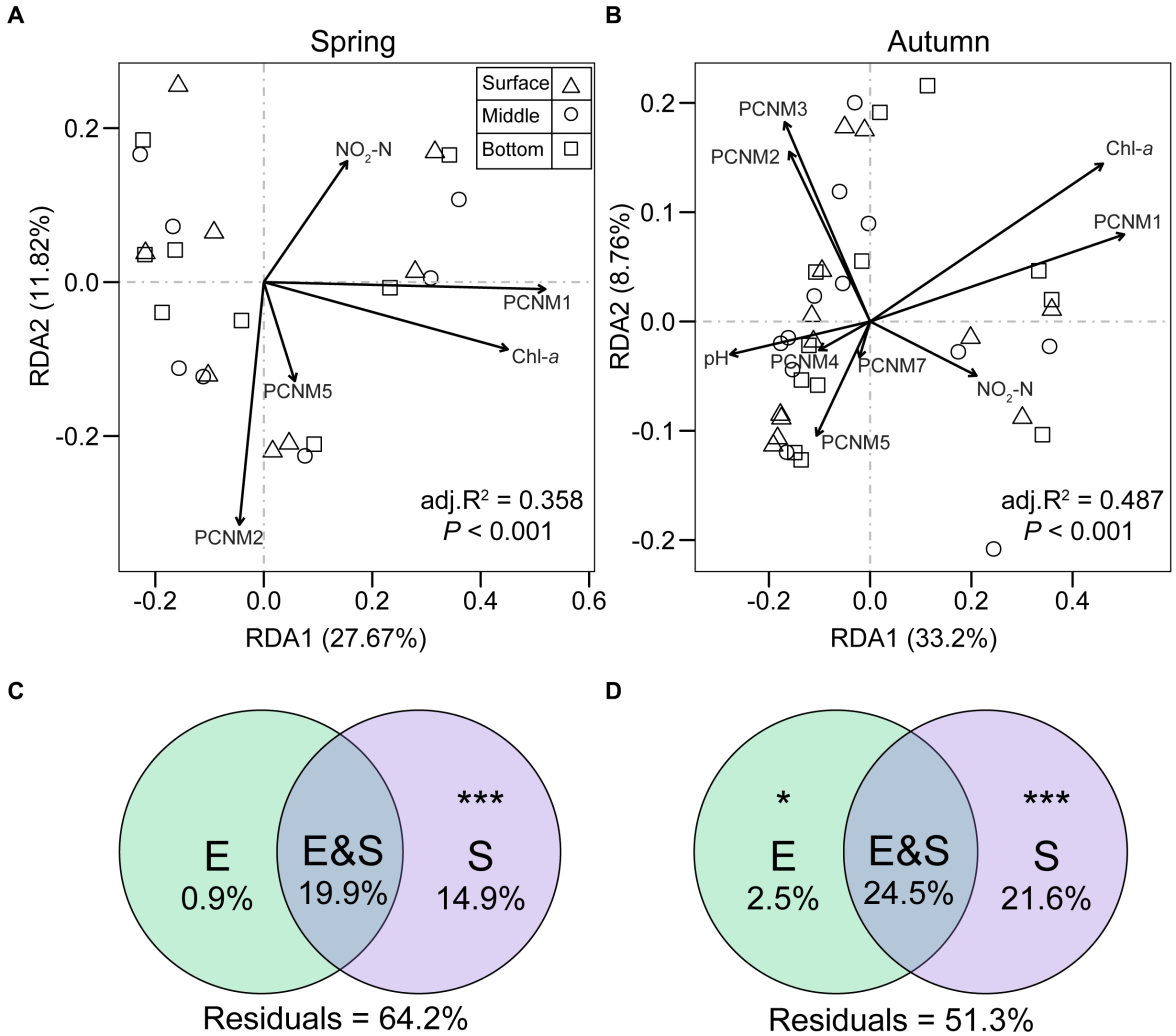
Redundancy analysis (RDA) and variation partitioning analysis (VPA) depict the effects of environmental and spatial (PCNM) factors on microeukaryotic communities variations during spring **(A,C)** and autumn **(B,D)** in Qianxia Lake. “E” represents relative contribution of environmental factors to community variation; “S” indicates relative contribution of spatial factors to community variation; “E&S” represents the shared explained variation; “Residuals” denote unexplained community variation. Statistical significance was assessed through permutational ANOVA (*p < 0.05 and ***p < 0.001).

RDA demonstrated variations in the percentage of explained variance for microeukaryotic communities by the first two axes across seasons, amounting to 39.5, 41.96, and 41.18% for spring, autumn, and both seasons, respectively (Figures 9A,B; Supplementary Figure S4). Environmental (Chl-*a* and NO_2_-N) and spatial variables (PCNM1, PCNM2, and PCNM5) exerted notable impacts on microeukaryotic community composition in both seasons by forward selection. In autumn, pH, PCNM3, PCNM4, and PCNM7 also showed significant correlations with communities. Variation partitioning analysis (VPA, Figures 9C,D) indicated that purely spatial variation played a larger role in community composition during spring (14.9%), autumn (21.6%), and both seasons (34.3%) compared to purely environmental factors (0.9% in spring, 2.5% in autumn, 9.3% in both seasons). Additionally, more than half of the proportion of community variation for each and both seasons was unexplained (51.3–64.2%, Figures 9C,D; Supplementary Figure S4).

**Figure 10:**
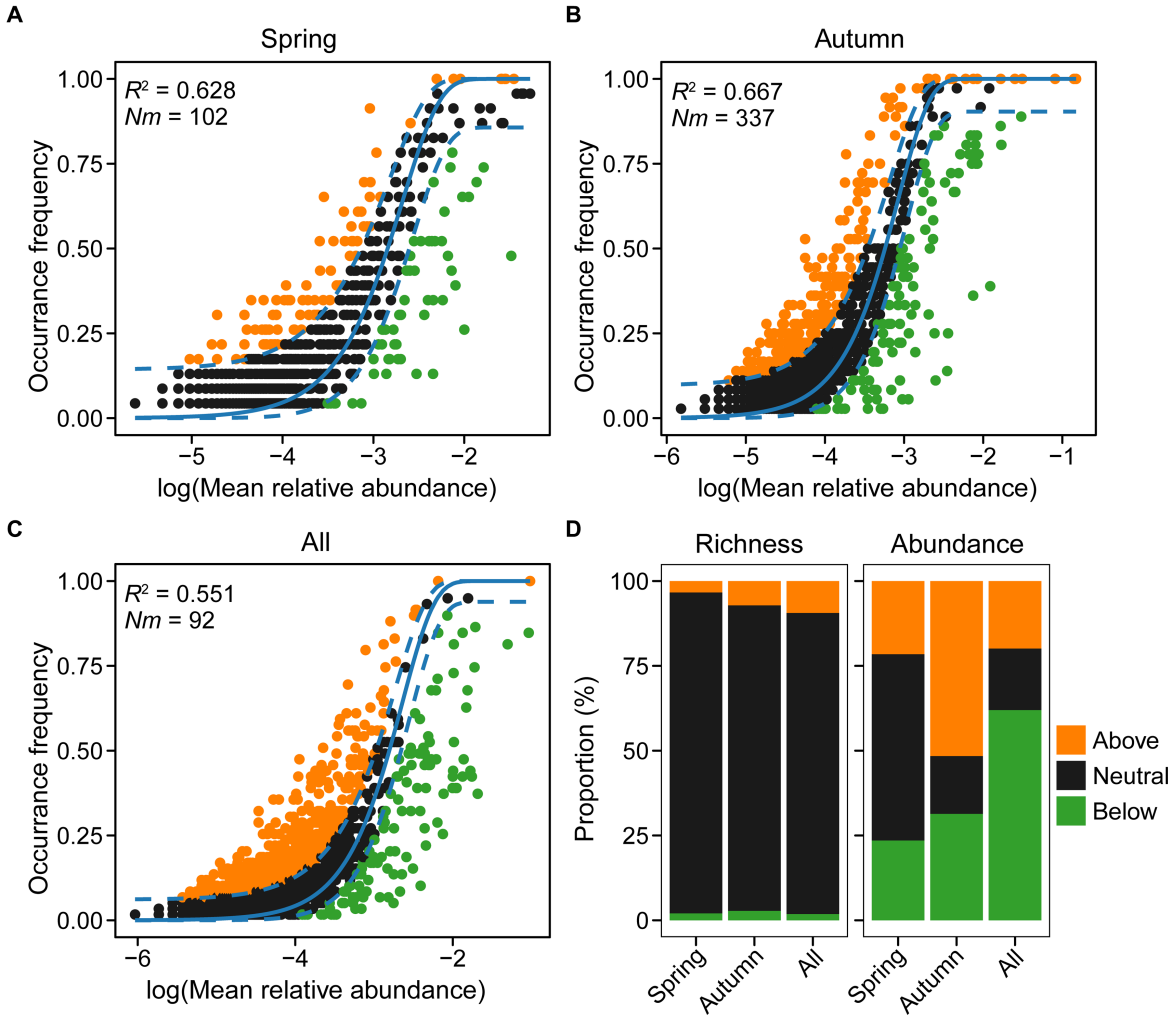
Predicted occurrence frequencies of amplicon sequence variants (ASVs) plotted against mean relative abundance, representing microeukaryotic communities in spring **(A)**, autumn **(B)**, and both seasons **(C)**, respectively. Variations in proportions of neutral and non-neutral partitions in relation to microeukaryotic richness and abundance **(D)**. The *Nm* value is determined by multiplying the metacommunity size (*N*) with immigration rate (m). The *R*^2^ value indicates the fit adequacy to the neutral model.

To assess the influence of stochastic processes on the assembly of microeukaryotic communities during spring and autumn, we employed the neutral community model (NCM). Results demonstrated that the NCM explained a substantial fraction (62.8, 66.7, and 55.1%) of variation in community composition for spring, autumn, and both seasons combined, respectively (Figure 10). This underscores the critical role of stochastic processes in influencing the assembly of microeukaryotic communities. The *N_m_* value for microeukaryotic taxa was lower in spring (*N_m_* = 102) than in autumn (*N_m_* = 337), indicating that higher species dispersal in autumn (*m*: 1.84% vs. 0.56%), due to sequence depth that was rarefied for both seasons (*N* = 18,300). Furthermore, the proportions of microeukaryotic ASVs revealed that the neutral fraction contributed significantly more to species richness (88.7–94.6%) than the above and below fractions in both seasons, but not to ASVs’ abundance (17–54.9%; Figure 10D). The findings, along with VPA results, suggest that stochastic processes primarily influenced community structure.

## Discussion

### Strong seasonality of planktonic microeukaryotic communities with negligible depth influence

The nMDS plot distinctly revealed the separation of samples between spring and autumn, indicating significant seasonality in planktonic microeukaryotic composition (*p* < 0.05) (Table 1; Figure 3). The seasonal differentiation can be attributed to variations in physicochemical factors, biotic interplays, and hydrologic environment (Sommer et al., 2012). Firstly, we observed significant differences in specific environmental factors (temperature, DO, pH) and nutrients (NO_2_-N, PO_4_-P, TP, and TOC) between the two seasons (Supplementary Table S2). Water temperature, a key seasonal factor, may directly regulate microeukaryotic community composition (Liu et al., 2013). Prior investigations indicated that elevated water temperatures could enhance the proliferation of microeukaryotic species (Liu et al., 2020), partially explaining the higher species richness in the autumn compared to spring (Figure 1A). Nitrite (NO_2_-N) emerged as the sole nutrient significantly co-varied with microeukaryotic community structure during both seasons, as revealed by RDA ordination (Figures 9A,B). Nitrite is a vital form of inorganic nitrogen readily assimilated by phytoplankton. Certain phytoplankton populations have been reported to consider nitrite as a more important nitrogen source than nitrate (Yves, 1998). Hence, nitrite concentration could mediate the phytoplankton community variation, thereby indirectly influencing the entire microeukaryotic community composition within the lake system. Secondly, the seasonal dynamics of microbial plankton community represent a repeatedly recurring process primarily influenced by environmental fluctuations and internal interactions, such as competition and predation (Sommer et al., 2012). Thirdly, the strong seasonality could also be explained by the high turnover of microeukaryotic communities (Zhang et al., 2017), with stochastic processes (ecological drift and dispersal) playing a significant role in structuring their communities (Logares et al., 2018).

The spring-autumn variations in microeukaryotic community composition were mainly characterized by a higher abundance of Dinoflagellata, Basidiomycota, Mesomycetozoa, and Apicomplexa in spring, while Chlorophyta, Haptista, and Choanoflagellida were more abundant in autumn (Figure 4; Supplementary Table S5). It is worth noting that the protists, such as ciliates, dinoflagellates, and fungi, possess much higher SSU rDNA copies than other protists, which could potentially result in an overestimation of their prevalence in environmental surveys (Godhe et al., 2008; Gong et al., 2013). Variations in single-celled rDNA copy number have been tightly linked with cell size, thus it is more appropriate to interpret the rDNA reads in relation to relative proportion of eukaryotic biomass (Fu and Gong, 2017; Zou S. et al., 2021). Therefore, planktonic dinoflagellates and fungi (Basidiomycota and Mesomycetozoa) might bloom in spring, resulting in higher biomass during this season.

Limited variability was noted in planktonic microeukaryotic communities across water column depths (Figure 3A; Table 1), despite there being significant differences in certain environmental variables (e.g., DO and temperature; Supplementary Table S2). This observation aligns with a previous study on bacterioplankton communities in a reservoir with a depth range of 0.5–20 m (Zlatković et al., 2022) but contrasts with research covering a much wider depth range. For example, Gong et al. (2015) documented that structure of microeukaryotic community was significantly shaped by depth in sediments of coastal shallow water (15–75 m). In addition, David et al. (2021) investigated the protistan community in Lake Baikal, Russia, and demonstrated a strong significant effect of depth (5–1,400 m) on protist community stratification. The limited variation in planktonic microbial communities within the epilimnion zone (0–20 m) of water column is likely due to wind-induced water mixing, which reduce stratification and constrain niche differentiation opportunities for microorganisms. Therefore, the homogeneity of microeukaryotic plankton can be understood as breakdown of stratification in the upper layer of water column, may have the following ecological consequences: (1) Upbringing nutrient-rich deeper waters to the surface, increasing the nutrient availability for diverse microbial groups and homogenizing environmental conditions (e.g., temperature, pH); (2) Promoting oxygenation to deeper hypoxic zones, stimulating the planktonic metabolic activities; (3) Facilitating the movement of microorganisms, increasing gene flow, genetic diversity, and the potential colonization of new habitats; and (4) Temporal changes in microbial community structure caused by the disruption of stratification, understanding these dynamics can help us gain insights into the resilience and adaptability of microbial communities.

### Species richness-productivity relationship in lake microeukaryotic communities follows a significant hump-shaped pattern

The species richness and productivity relationships (SRPR) at regional and local scales are long-standing and fundamental aspects of ecological research. SRPR can exhibit various patterns, such as linear, unimodal, U-shaped, or non-significant patterns (Waide et al., 1999; Mittelbach et al., 2001; Whittaker and Heegaard, 2003; Wang et al., 2020). Among these patterns, hump-shaped relationships occur most frequently, particularly in aquatic systems (Mittelbach et al., 2001). Our current study demonstrated a hump-shaped curve between microeukaryotic diversity and Chl-*a* (Figures 1B,D), and this pattern was mainly attributed to Dinoflagellata, Basidiomycota, Oomycota, and Perkinsozoa, most of which were heterotrophs, with the exception of Dinoflagellata (Supplementary Figure S2; Supplementary Table S4). Our observations of microeukaryotic species richness maximizing at intermediate productivity levels (~4.0 μgL^−1^) align with earlier research in marine (Spatharis et al., 2008) and lake ecosystems (Dodson et al., 2000; Grover and Chrzanowski, 2004). Several hypotheses have been proposed based on existing ecological theories to explain the hump-shaped relationship. At the low productivity zone (left end of the curve), species richness seems primarily influenced by nutrient limitation (Spatharis et al., 2007). As productivity increases, the abundance of rare species may surge, leading to increased species richness (Abrams, 1995). Nonetheless, within the high productivity regime (rightmost of the curve), a reduction in species richness may be due to increased competition or severe environmental stressors (Dodson et al., 2000; Spatharis et al., 2008). These findings underscore the complex ecological dynamics that govern microeukaryotic biodiversity patterns in aquatic ecosystems.

### Linking functional shifts of microeukaryotic planktons to environmental drivers

The diversity and taxonomic composition of communities exhibited significant correlations and formed hump-shaped relationships with Chl-*a* and DO (Figures 1B,D, 3D; Supplementary Figure S2). However, no similar patterns were observed for phototrophs and heterotrophs, except for mixotrophs when using trait-based functional analysis (Figure 6C). The decoupling between taxonomic and functional diversity contrasts with the findings of Ramond et al. (2019), who indicated a strong association between the functional diversity of marine protist communities and their taxonomic diversity across different size fractions. The discrepancy might be ascribed to their transcontinental survey (~3,800 km) in contrast to our localized one (40 km). Studies on microbial biogeography and large-scale environmental surveys have documented higher diversity with larger sampling scales, potentially leading to increased functional diversity (Martiny et al., 2006). Additionally, in comparison to the various traits annotated by Ramond et al. (2019), we acknowledge limitations in simplifying to three functional traits.

Random forest analysis indicated that the alterations in functional composition of freshwater microeukaryotic communities result from various environmental factors (i.e., N:P ratio, Chl-*a*, DO, TP, and TOC; Figure 8), which are broadly consistent with findings from several recent investigations (Amorim and Moura, 2021; Lu et al., 2023; Xue et al., 2023). The determined nutrient levels showed relatively high contents of PO_4_-P (on average 0.14 mg/L) but low levels of DIN (dominated by NO_3_-N, 0.56 mg/L) in the regime of Qianxia Lake. Furthermore, the mean N:P ratio of 4.6:1 was significantly below the Redfield ratio of 16:1. Collectively, these results indicate an N-deficit condition within the studied region. The N limitation may be one of the reasons for both phototrophic and mixotrophic abundance positively correlated with the N:P ratio (Figure 8B). Under N-deficit condition, the elevated prevalence of heterotrophs could be explained by heterotrophic microeukaryotes obtaining nutrients by grazing bacteria (Sherr and Sherr, 2002). The stronger relationship of relative proportion with Chl-*a* rather than DO in this study suggests that productivity probably plays a more crucial role than DO in indicating the abundance of mixotrophs. This finding, however, contrasts with our previous investigation of pico−/nanoeukaryotes in coastal oceans (Wang et al., 2020).

### Significant distance-decay relationship suggests pronounced dispersal limitation effects

In the current investigation, a notable distance-decay pattern of the microeukaryotic community was observed in Qianxia Lake of Southeast China (Figures 3B,C). The calculated turnover rate (*z* value) for microeukaryotes in our research was 0.014, falling within the range (0.0019 ~ 0.26) documented for microbial communities by Woodcock et al. (2006). This pronounced turnover in biodiversity is typically ascribed to a dynamic balance between extinction and immigration, coupled with an increase in habitat diversity over greater spatial distances (Kallimanis et al., 2008). Previous microbial biogeographic investigations have disclosed a decline within certain populations over distance, highlighting the substantial influence of dispersal limitation and resulting in conspicuous regional uniqueness (Martiny et al., 2006; Isabwe et al., 2018; Bai et al., 2022). This challenges the microbial ubiquitous dispersal hypothesis (Finlay and Clarke, 1999). Given the potential impact of both deterministic and stochastic factors on dispersion, dispersal limitation could theoretically be influenced by stochastic, deterministic, or both processes. Nevertheless, it is commonly accepted that dispersal limitation is a neutral stochastic process (Hubbell, 2001; Zhou and Ning, 2017).

### Stochastic processes predominate the assembly of microeukaryotic communities

The neutral community model revealed the significant influence of stochastic processes on the assembly of microeukaryotic communities (Figure 10). Based on the robust assumption of ecological equivalence (Hubbell, 2005), this theory allows quantification of elusive processes—birth or death, dispersal, speciation, and ecological drift. The model has been widely applied to elucidate various ecological phenomena due to its simplicity and predictability (Heys et al., 2020; Jiao et al., 2020). The NCM explained a significant portion of microeukaryotic community variation across seasons, with over 63% of variation for each season and over 55% in total (Figure 10). This implies a stronger impact of stochastic processes on microeukaryotic communities compared to deterministic processes. The dominance of stochastic processes was reinforced by the results of VPA (Figure 9), which demonstrated that spatial factors contribute more to shaping microeukaryotic communities than environmental factors. These findings are consistent with previous research, for instance, Chen et al. (2019) revealed that microeukaryotic communities in river ecosystem were mainly shaped by stochastic processes, with 88.5 to 89.9% of the community variation explained by NCM. Similarly, in a study of Zou K. et al. (2021) investigating protist communities assembly in the Pearl River Delta with serious anthropogenic disturbance, NCM accounted for 51.24% ~ 75.82% of community variation. These findings collectively demonstrated that the assembly of freshwater microbial eukaryotic plankton was primarily driven by stochastic processes.

Variance partitioning analysis (VPA) is a valuable statistical approach extensively employed for evaluating the importance of environmental and spatial factors in the variation of microbial community ecology (Wu et al., 2020; Jing et al., 2023). Regrettably, our VPA models revealed that over 50% of the community variation remained unaccounted for (Figure 9). This observation aligns with several previous studies on microeukaryotic biogeography (Chen et al., 2019; Zhu et al., 2021), suggesting a limited impact of environmental and spatial factors on the microeukaryotic community. The large unexplained proportion could be due to influential factors not detected and included in the VPA. Furthermore, the inherent complexity of microbial ecological systems with intricate interactions, such as synergistic or antagonistic effects, poses challenges that may not be easily addressed by VPA (Meidute et al., 2008). It is also noteworthy that VPA tends to exaggerate the impact of spatial factors (Gilbert and Bennett, 2010). Consequently, enhancing the explanatory capacity of VPA may necessitate a broader scope of data acquisition, and the exploration of alternative modeling strategies, such as incorporating neutral community model, to ameliorate the inherent limitations of VPA.

## Data availability statement

The data acquired in this investigation have been uploaded to the NCBI Sequence Read Archive (SRA) database, accessible through BioProject PRJNA952349 with accession number SRR24098043-SRR24098102.

## Author contributions

SZ: Conceptualization, Data curation, Formal analysis, Investigation, Methodology, Resources, Software, Validation, Visualization, Writing – original draft, Writing – review & editing. QL: Funding acquisition, Investigation, Project administration, Writing – original draft. MN: Methodology, Writing – original draft. DZ: Investigation, Writing – original draft. ML: Writing – original draft. XZ: Investigation, Writing – original draft. GC: Resources, Writing – review & editing, Funding acquisition. JY: Funding acquisition, Project administration, Supervision, Writing – review & editing.

## References

[ref1] Abdullah Al M. Xue Y. Xiao P. Xu J. Chen H. Mo Y.. (2022). Community assembly of microbial habitat generalists and specialists in urban aquatic ecosystems explained more by habitat type than pollution gradient. Water Res. 220:118693. doi: 10.1016/j.watres.2022.118693, PMID: 35667165

[ref2] Abrams P. A. (1995). Monotonic or unimodal diversity-productivity gradients: what does competition theory predict? Ecology 76, 2019–2027. doi: 10.2307/1941677

[ref3] Adl S. M. Bass D. Lane C. E. Lukeš J. Schoch C. L. Smirnov A.. (2019). Revisions to the classification, nomenclature, and diversity of eukaryotes. J. Eukaryot. Microbiol. 66, 4–119. doi: 10.1111/jeu.12691, PMID: 30257078 PMC6492006

[ref4] Amorim C. A. Moura A. D. N. (2021). Ecological impacts of freshwater algal blooms on water quality, plankton biodiversity, structure, and ecosystem functioning. Sci. Total Environ. 758:143605. doi: 10.1016/j.scitotenv.2020.143605, PMID: 33248793

[ref5] Anderson M. J. (2001). A new method for non-parametric multivariate analysis of variance. Austral Ecol. 26, 32–46. doi: 10.1111/j.1442-9993.2001.01070.pp.x

[ref6] Archer E. (2019). rfPermute: estimate permutation *p*-values for random Forest importance metrics. R Package Version 2.5.2. Vienna: R Foundation for Statistical Computing.

[ref7] Azam F. Malfatti F. (2007). Microbial structuring of marine ecosystems. Nat. Rev. Microbiol. 5, 782–791. doi: 10.1038/nrmicro1747 17853906

[ref8] Bai C. Gao G. Tang X. Shao K. Hu Y. Xia J.. (2022). Contrasting diversity patterns and community assembly mechanisms of bacterioplankton among different aquatic habitats in Lake Taihu, a large eutrophic shallow lake in China. Environ. Pollut. 315:120342. doi: 10.1016/j.envpol.2022.120342, PMID: 36240961

[ref9] Blanchet F. G. Legendre P. Borcard D. (2008). Forward selection of explanatory variables. Ecology 89, 2623–2632. doi: 10.1890/07-0986.1 18831183

[ref10] Borcard D. Legendre P. (2002). All-scale spatial analysis of ecological data by means of principal coordinates of neighbour matrices. Ecol. Model. 153, 51–68. doi: 10.1016/S0304-3800(01)00501-4

[ref11] Borcard D. Legendre P. Drapeau P. (1992). Partialling out the spatial component of ecological variation. Ecology 73, 1045–1055. doi: 10.2307/1940179

[ref12] Borics G. Görgényi J. Grigorszky I. László-Nagy Z. Tóthmérész B. Krasznai E.. (2014). The role of phytoplankton diversity metrics in shallow lake and river quality assessment. Ecol. Indic. 45, 28–36. doi: 10.1016/j.ecolind.2014.03.011

[ref13] Callahan B. J. McMurdie P. J. Rosen M. J. Han A. W. Johnson A. J. A. Holmes S. P. (2016). DADA2: high-resolution sample inference from Illumina amplicon data. Nat. Methods 13, 581–583. doi: 10.1038/nmeth.3869, PMID: 27214047 PMC4927377

[ref14] Chambers J. M. (2008). Software for data analysis: programming with R. Springer, New York City, New York.

[ref15] Chase J. M. Leibold M. A. (2002). Spatial scale dictates the productivity–biodiversity relationship. Nature 416, 427–430. doi: 10.1038/416427a, PMID: 11919631

[ref16] Chave J. (2004). Neutral theory and community ecology. Ecol. Lett. 7, 241–253. doi: 10.1111/j.1461-0248.2003.00566.x

[ref17] Chen W. Ren K. Isabwe A. Chen H. Liu M. Yang J. (2019). Stochastic processes shape microeukaryotic community assembly in a subtropical river across wet and dry seasons. Microbiome 7:138. doi: 10.1186/s40168-019-0749-8, PMID: 31640783 PMC6806580

[ref18] Chesson P. (2000). Mechanisms of maintenance of species diversity. Annu. Rev. Ecol. Evol. Syst. 31, 343–366. doi: 10.1146/annurev.ecolsys.31.1.343

[ref19] David G. M. Moreira D. Reboul G. Annenkova N. V. Galindo L. J. Bertolino P.. (2021). Environmental drivers of plankton protist communities along latitudinal and vertical gradients in the oldest and deepest freshwater lake. Environ. Microbiol. 23, 1436–1451. doi: 10.1111/1462-2920.15346, PMID: 33270368

[ref20] Dodson S. I. Arnott S. E. Cottingham K. L. (2000). The relationship in lake communities between primary productivity and species richness. Ecology 81, 2662–2679. doi: 10.1890/0012-9658(2000)081[2662:TRILCB]2.0.CO;2

[ref21] Fargione J. Brown C. S. Tilman D. (2003). Community assembly and invasion: An experimental test of neutral versus niche processes. Proc. Natl. Acad. Sci. USA 100, 8916–8920. doi: 10.1073/pnas.1033107100, PMID: 12843401 PMC166413

[ref22] Finlay B. J. Clarke K. J. (1999). Ubiquitous dispersal of microbial species. Nature 400:828. doi: 10.1038/23616

[ref23] Foissner W. Berger H. (1996). A user-friendly guide to the ciliates (Protozoa, Ciliophora) commonly used by hydrobiologists as bioindicators in rivers, lakes, and waste waters, with notes on their ecology. Freshw. Biol. 35, 375–482. doi: 10.1111/j.1365-2427.1996.tb01775.x

[ref24] Fortmann-Roe S. (2015). Consistent and clear reporting of results from diverse modeling techniques: the A3 method. J. Stat. Softw. 66, 1–23. doi: 10.18637/jss.v066.i07

[ref25] Fu R. Gong J. (2017). Single cell analysis linking ribosomal (r)DNA and rRNA copy numbers to cell size and growth rate provides insights into molecular protistan ecology. J. Eukaryot. Microbiol. 64, 885–896. doi: 10.1111/jeu.12425, PMID: 28499076 PMC5697653

[ref26] Gad M. Hou L. Cao M. Adyari B. Zhang L. Qin D.. (2022). Tracking microeukaryotic footprint in a peri-urban watershed, China through machine-learning approaches. Sci. Total Environ. 806:150401. doi: 10.1016/j.scitotenv.2021.150401, PMID: 34562761

[ref27] Gad M. Hou L. Li J. Wu Y. Rashid A. Chen N.. (2020). Distinct mechanisms underlying the assembly of microeukaryotic generalists and specialists in an anthropogenically impacted river. Sci. Total Environ. 748:141434. doi: 10.1016/j.scitotenv.2020.141434, PMID: 32814298

[ref28] Genitsaris S. Monchy S. Viscogliosi E. Sime-Ngando T. Ferreira S. Christaki U. (2015). Seasonal variations of marine protist community structure based on taxon-specific traits using the eastern English Channel as a model coastal system. FEMS Microbiol. Ecol. 91:fiv034. doi: 10.1093/femsec/fiv034, PMID: 25873460

[ref29] Gilbert B. Bennett J. R. (2010). Partitioning variation in ecological communities: do the numbers add up? J. Appl. Ecol. 47, 1071–1082. doi: 10.1111/j.1365-2664.2010.01861.x

[ref30] Godhe A. Asplund Maria E. Härnström K. Saravanan V. Tyagi A. Karunasagar I. (2008). Quantification of diatom and dinoflagellate biomasses in coastal marine seawater samples by real-time PCR. Appl. Environ. Microbiol. 74, 7174–7182. doi: 10.1128/AEM.01298-08, PMID: 18849462 PMC2592920

[ref31] Gong J. Dong J. Liu X. Massana R. (2013). Extremely high copy numbers and polymorphisms of the rDNA operon estimated from single cell analysis of oligotrich and peritrich ciliates. Protist 164, 369–379. doi: 10.1016/j.protis.2012.11.006, PMID: 23352655

[ref32] Gong J. Shi F. Ma B. Dong J. Pachiadaki M. Zhang X.. (2015). Depth shapes α-and β-diversities of microbial eukaryotes in surficial sediments of coastal ecosystems. Environ. Microbiol. 17, 3722–3737. doi: 10.1111/1462-2920.12763, PMID: 25581721

[ref33] Grover J. P. Chrzanowski T. H. (2004). Limiting resources, disturbance, and diversity in phytoplankton communities. Ecol. Monogr. 74, 533–551. doi: 10.1890/03-4073

[ref34] Han M. Huang J. Yang J. Wang B. Sun X. Jiang H. (2023). Distinct assembly mechanisms for prokaryotic and microeukaryotic communities in the water of Qinghai Lake. J. Earth Sci. 34, 1189–1200. doi: 10.1007/s12583-023-1812-8

[ref35] Heys C. Cheaib B. Busetti A. Kazlauskaite R. Maier L. Sloan W. T.. (2020). Neutral processes dominate microbial community assembly in Atlantic salmon, *Salmo salar*. Appl. Environ. Microbiol. 86, e02283–e02219. doi: 10.1128/AEM.02283-19, PMID: 32033945 PMC7117918

[ref36] Hijmans R. J. Williams E. Vennes C. Hijmans M. R. J. (2017). Package “geosphere”: spherical trigonometry. R Package Version 1.5-18. Vienna: R Foundation for Statistical Computing.

[ref37] Hou F. Zhang H. Xie W. Zhou X. Zhu X. Zhang D. (2020). Co-occurrence patterns and assembly processes of microeukaryotic communities in an early-spring diatom bloom. Sci. Total Environ. 711:134624. doi: 10.1016/j.scitotenv.2019.134624, PMID: 31818596

[ref38] Hubbell S. P. (2001). The unified neutral theory of biodiversity and biogeography. Princeton, NJ; Oxford, UK: Princeton University Press.

[ref39] Hubbell S. P. (2005). Neutral theory in community ecology and the hypothesis of functional equivalence. Funct. Ecol. 19, 166–172. doi: 10.1111/j.0269-8463.2005.00965.x

[ref40] Hulatt C. J. Thomas D. N. (2010). Dissolved organic matter (DOM) in microalgal photobioreactors: a potential loss in solar energy conversion? Bioresour. Technol. 101, 8690–8697. doi: 10.1016/j.biortech.2010.06.086, PMID: 20634058

[ref41] Isabwe A. Ren K. Wang Y. Peng F. Chen H. Yang J. (2019). Community assembly mechanisms underlying the core and random bacterioplankton and microeukaryotes in a river-reservoir system. Water 11:1127. doi: 10.3390/w11061127

[ref42] Isabwe A. Yang J. R. Wang Y. Liu L. Chen H. Yang J. (2018). Community assembly processes underlying phytoplankton and bacterioplankton across a hydrologic change in a human-impacted river. Sci. Total Environ. 630, 658–667. doi: 10.1016/j.scitotenv.2018.02.210, PMID: 29494974

[ref43] Jiao S. Yang Y. Xu Y. Zhang J. Lu Y. (2020). Balance between community assembly processes mediates species coexistence in agricultural soil microbiomes across eastern China. ISME J. 14, 202–216. doi: 10.1038/s41396-019-0522-9, PMID: 31611655 PMC6908645

[ref44] Jing M. Yang W. Ding X. Rao L. Zhang Q. Zhu J. (2023). Environmental heterogeneity associated with boat activity shapes bacteria and microeukaryotic communities with discrepant response patterns. Sci. Total Environ. 903:166943. doi: 10.1016/j.scitotenv.2023.166943, PMID: 37690748

[ref45] Kallimanis A. S. Mazaris A. D. Tzanopoulos J. Halley J. M. Pantis J. D. Sgardelis S. P. (2008). How does habitat diversity affect the species–area relationship? Glob. Ecol. Biogeogr. 17, 532–538. doi: 10.1111/j.1466-8238.2008.00393.x

[ref46] Liu J. Meng Z. Liu X. Zhang X.-H. (2019). Microbial assembly, interaction, functioning, activity and diversification: a review derived from community compositional data. Mar. Life Sci. Technol. 1, 112–128. doi: 10.1007/s42995-019-00004-3

[ref47] Liu Y. Ren Z. Qu X. Zhang M. Yu Y. Peng W. (2020). Seasonal water level fluctuation and concomitant change of nutrients shift microeukaryotic communities in a shallow lake. Water 12:2317. doi: 10.3390/w12092317

[ref48] Liu L. Yang J. Yu X. Chen G. Yu Z. (2013). Patterns in the composition of microbial communities from a subtropical river: effects of environmental, spatial and temporal factors. PLoS One 8:e81232. doi: 10.1371/journal.pone.0081232, PMID: 24244735 PMC3828266

[ref49] Logares R. Deutschmann I. M. Junger P. C. Giner C. R. Krabberød A. K. Schmidt T. S. B.. (2020). Disentangling the mechanisms shaping the surface ocean microbiota. Microbiome 8:55. doi: 10.1186/s40168-020-00827-8, PMID: 32312331 PMC7171866

[ref50] Logares R. Lindström E. S. Langenheder S. Logue J. B. Paterson H. Laybourn-Parry J.. (2013). Biogeography of bacterial communities exposed to progressive long-term environmental change. ISME J. 7, 937–948. doi: 10.1038/ismej.2012.168, PMID: 23254515 PMC3635229

[ref51] Logares R. Tesson S. V. M. Canbäck B. Pontarp M. Hedlund K. Rengefors K. (2018). Contrasting prevalence of selection and drift in the community structuring of bacteria and microbial eukaryotes. Environ. Microbiol. 20, 2231–2240. doi: 10.1111/1462-2920.14265, PMID: 29727053

[ref52] Lu X. Lv B. Han Y. Tian W. Jiang T. Zhu G.. (2023). Responses of compositions, functions, and assembly processes of bacterial and microeukaryotic communities to long-range voyages in simulated ballast water. Mar. Environ. Res. 190:106115. doi: 10.1016/j.marenvres.2023.106115, PMID: 37540963

[ref53] Martin M. (2011). Cutadapt removes adapter sequences from high-throughput sequencing reads. EMBnet J. 17:3. doi: 10.14806/ej.17.1.200

[ref54] Martiny J. B. H. Bohannan B. J. M. Brown J. H. Colwell R. K. Fuhrman J. A. Green J. L.. (2006). Microbial biogeography: putting microorganisms on the map. Nat. Rev. Microbiol. 4, 102–112. doi: 10.1038/nrmicro1341, PMID: 16415926

[ref55] McMurdie P. J. Holmes S. (2014). Waste not, want not: why rarefying microbiome data is inadmissible. PLoS Comput. Biol. 10:e1003531. doi: 10.1371/journal.pcbi.1003531, PMID: 24699258 PMC3974642

[ref56] Meidute S. Demoling F. Bååth E. (2008). Antagonistic and synergistic effects of fungal and bacterial growth in soil after adding different carbon and nitrogen sources. Soil Biol. Biochem. 40, 2334–2343. doi: 10.1016/j.soilbio.2008.05.011

[ref57] Mittelbach G. G. Steiner C. F. Scheiner S. M. Gross K. L. Reynolds H. L. Waide R. B.. (2001). What is the observed relationship between species richness and productivity? Ecology 82, 2381–2396. doi: 10.1890/0012-9658(2001)082[2381:WITORB]2.0.CO;2

[ref58] Mo Y. Peng F. Gao X. Xiao P. Logares R. Jeppesen E.. (2021). Low shifts in salinity determined assembly processes and network stability of microeukaryotic plankton communities in a subtropical urban reservoir. Microbiome 9:128. doi: 10.1186/s40168-021-01079-w, PMID: 34082826 PMC8176698

[ref59] Nemergut D. R. Schmidt S. K. Fukami T. O'Neill S. P. Bilinski T. M. Stanish L. F.. (2013). Patterns and processes of microbial community assembly. Microbiol. Mol. Biol. Rev. 77, 342–356. doi: 10.1128/mmbr.00051-12, PMID: 24006468 PMC3811611

[ref60] Oksanen J. Kindt R. Legendre P. O’Hara B. Stevens M. H. H. Oksanen M. J.. (2013). Vegan: community ecology package. R Package Version 2.6-2. Vienna: R Foundation for Statistical Computing.

[ref61] Pan Y. Li G. Su L. Zheng P. Wang Y. Shen Z.. (2022). Seagrass colonization alters diversity, abundance, taxonomic, and functional community structure of benthic microbial eukaryotes. Front. Microbiol. 13:901741. doi: 10.3389/fmicb.2022.901741, PMID: 35770161 PMC9234489

[ref62] R Core Team (2018). R: a language and environment for statistical computing. Vienna: R Foundation for Statistical computing.

[ref63] Ramond P. Sourisseau M. Simon N. Romac S. Schmitt S. Rigaut-Jalabert F.. (2019). Coupling between taxonomic and functional diversity in protistan coastal communities. Environ. Microbiol. 21, 730–749. doi: 10.1111/1462-2920.14537, PMID: 30672084

[ref64] Ranjard L. Dequiedt S. Chemidlin Prévost-Bouré N. Thioulouse J. Saby N. P. A. Lelievre M.. (2013). Turnover of soil bacterial diversity driven by wide-scale environmental heterogeneity. Nat. Commun. 4:1434. doi: 10.1038/ncomms2431, PMID: 23385579

[ref65] Ren Z. Ma K. Jia X. Wang Q. Zhang C. Li X. (2022). Community assembly and co-occurrence patterns of microeukaryotes in Thermokarst Lakes of the Yellow River source area. Microorganisms 10:481. doi: 10.3390/microorganisms10020481, PMID: 35208934 PMC8877526

[ref66] Righetti D. Vogt M. Gruber N. Psomas A. Zimmermann N. E. (2019). Global pattern of phytoplankton diversity driven by temperature and environmental variability. Sci. Adv. 5:eaau6253. doi: 10.1126/sciadv.aau6253, PMID: 31106265 PMC6520023

[ref67] Roberts D. W. (2019). Labdsv: ordination and multivariate analysis for ecology. R Package Version 2.1-0. Vienna: R Foundation for Statistical Computing.

[ref68] Robinson M. D. McCarthy D. J. Smyth G. K. (2010). edgeR: a Bioconductor package for differential expression analysis of digital gene expression data. Bioinformatics 26, 139–140. doi: 10.1093/bioinformatics/btp616, PMID: 19910308 PMC2796818

[ref69] Sherr E. B. Sherr B. F. (2002). Significance of predation by protists in aquatic microbial food webs. Antonie Van Leeuwenhoek 81, 293–308. doi: 10.1023/A:1020591307260, PMID: 12448728

[ref70] Sloan W. T. Lunn M. Woodcock S. Head I. M. Nee S. Curtis T. P. (2006). Quantifying the roles of immigration and chance in shaping prokaryote community structure. Environ. Microbiol. 8, 732–740. doi: 10.1111/j.1462-2920.2005.00956.x, PMID: 16584484

[ref71] Sommer U. Adrian R. Domis L. D. S. Elser J. J. Gaedke U. Ibelings B.. (2012). Beyond the plankton ecology group (PEG) model: mechanisms driving plankton succession. Annu. Rev. Ecol. Evol. Syst. 43, 429–448. doi: 10.1146/annurev-ecolsys-110411-160251

[ref72] Spatharis S. Mouillot D. Danielidis D. B. Karydis M. Chi T. D. Tsirtsis G. (2008). Influence of terrestrial runoff on phytoplankton species richness-biomass relationships: a double stress hypothesis. J. Exp. Mar. Biol. Ecol. 362, 55–62. doi: 10.1016/j.jembe.2008.06.003

[ref73] Spatharis S. Tsirtsis G. Danielidis D. B. Chi T. D. Mouillot D. (2007). Effects of pulsed nutrient inputs on phytoplankton assemblage structure and blooms in an enclosed coastal area. Estuar. Coast. Shelf Sci. 73, 807–815. doi: 10.1016/j.ecss.2007.03.016

[ref74] Stoeck T. Bass D. Nebel M. Christen R. Jones M. D. M. Breiner H.-W.. (2010). Multiple marker parallel tag environmental DNA sequencing reveals a highly complex eukaryotic community in marine anoxic water. Mol. Ecol. 19, 21–31. doi: 10.1111/j.1365-294X.2009.04480.x, PMID: 20331767

[ref75] Treuer G. Kirchhoff C. Lemos M. C. McGrath F. (2021). Challenges of managing harmful algal blooms in US drinking water systems. Nat. Sustain. 4, 958–964. doi: 10.1038/s41893-021-00770-y

[ref76] Vallina S. M. Follows M. J. Dutkiewicz S. Montoya J. M. Cermeno P. Loreau M. (2014). Global relationship between phytoplankton diversity and productivity in the ocean. Nat. Commun. 5:4299. doi: 10.1038/ncomms5299, PMID: 24980772 PMC4102128

[ref77] Waide R. B. Willig M. R. Steiner C. F. Mittelbach G. Gough L. Dodson S. I.. (1999). The relationship between productivity and species richness. Annu. Rev. Ecol. Evol. Syst. 30, 257–300. doi: 10.1146/annurev.ecolsys.30.1.257

[ref78] Wang Y. Li G. Shi F. Dong J. Gentekaki E. Zou S.. (2020). Taxonomic diversity of pico−/nanoeukaryotes is related to dissolved oxygen and productivity, but functional composition is shaped by limiting nutrients in eutrophic coastal oceans. Front. Microbiol. 11:601037. doi: 10.3389/fmicb.2020.601037, PMID: 33343542 PMC7744618

[ref79] Wang Y. Liu L. Chen H. Yang J. (2015). Spatiotemporal dynamics and determinants of planktonic bacterial and microeukaryotic communities in a Chinese subtropical river. Appl. Microbiol. Biotechnol. 99, 9255–9266. doi: 10.1007/s00253-015-6773-0, PMID: 26156239

[ref80] Whittaker R. J. Heegaard E. (2003). What is the observed relationship between species richness and productivity? Comment. Ecology 84, 3384–3390. doi: 10.1890/02-3128

[ref81] Woodcock S. Curtis T. P. Head I. M. Lunn M. Sloan W. T. (2006). Taxa–area relationships for microbes: the unsampled and the unseen. Ecol. Lett. 9, 805–812. doi: 10.1111/j.1461-0248.2006.00929.x, PMID: 16796570

[ref82] Wright E. S. (2016). Using DECIPHER v2. 0 to analyze big biological sequence data in R. R J. 8, 352–359. doi: 10.32614/rj-2016-025

[ref83] Wu P.-F. Li D.-X. Kong L.-F. Li Y.-Y. Zhang H. Xie Z.-X.. (2020). The diversity and biogeography of microeukaryotes in the euphotic zone of the northwestern Pacific Ocean. Sci. Total Environ. 698:134289. doi: 10.1016/j.scitotenv.2019.134289, PMID: 31514034

[ref84] Wu W. Lu H.-P. Sastri A. Yeh Y.-C. Gong G.-C. Chou W.-C.. (2018). Contrasting the relative importance of species sorting and dispersal limitation in shaping marine bacterial versus protist communities. ISME J. 12, 485–494. doi: 10.1038/ismej.2017.183, PMID: 29125596 PMC5776463

[ref85] Wu J. Zhu Z. Waniek J. J. Niu M. Wang Y. Zhang Z.. (2023). The biogeography and co-occurrence network patterns of bacteria and microeukaryotes in the estuarine and coastal waters. Mar. Environ. Res. 184:105873. doi: 10.1016/j.marenvres.2023.105873, PMID: 36628821

[ref86] Xu D. Sun P. Zhang Y. Li R. Huang B. Jiao N.. (2018). Pigmented microbial eukaryotes fuel the deep sea carbon pool in the tropical Western Pacific Ocean. Environ. Microbiol. 20, 3811–3824. doi: 10.1111/1462-2920.14396, PMID: 30159996

[ref87] Xue Y. Chen H. Xiao P. Jin L. Logares R. Yang J. (2023). Core taxa drive microeukaryotic community stability of a deep subtropical reservoir after complete mixing. Environ. Microbiol. Rep. 15, 769–782. doi: 10.1111/1758-2229.13196, PMID: 37688478 PMC10667671

[ref88] Yao Y. Gu Z. Li Y. Ding H. Wang T. (2022). Intelligent simulation of water temperature stratification in the reservoir. Int. J. Environ. Res. Public Health 19:13588. doi: 10.3390/ijerph192013588, PMID: 36294167 PMC9603658

[ref89] Yu Z. Yang J. Zhou J. Yu X. Liu L. Lv H. (2014). Water stratification affects the microeukaryotic community in a subtropical deep reservoir. J. Eukaryot. Microbiol. 61, 126–133. doi: 10.1111/jeu.12090, PMID: 24373024

[ref90] Yves C. (1998). Nitrate uptake, nitrite release and uptake, and new production estimates. Mar. Ecol. Prog. Ser. 171, 293–301. doi: 10.3354/meps171293

[ref91] Zhang H. Jia J. Chen S. Huang T. Wang Y. Zhao Z.. (2018). Dynamics of bacterial and fungal communities during the outbreak and decline of an algal bloom in a drinking water reservoir. Int. J. Environ. Res. Public Health 15:361. doi: 10.3390/ijerph15020361, PMID: 29463021 PMC5858430

[ref92] Zhang N. Xiao X. Pei M. Liu X. Liang Y. (2017). Discordant temporal turnovers of sediment bacterial and eukaryotic communities in response to dredging: nonresilience and functional changes. Appl. Environ. Microbiol. 83, e02526–e02516. doi: 10.1128/AEM.02526-16, PMID: 27793828 PMC5165113

[ref93] Zhou J. Ning D. (2017). Stochastic community assembly: does it matter in microbial ecology? Microbiol. Mol. Biol. Rev. 81:e00002-17. doi: 10.1128/mmbr.00002-17, PMID: 29021219 PMC5706748

[ref94] Zhu C. Liu W. Li X. Xu Y. El-Serehy H. A. Al-Farraj S. A.. (2021). High salinity gradients and intermediate spatial scales shaped similar biogeographical and co-occurrence patterns of microeukaryotes in a tropical freshwater-saltwater ecosystem. Environ. Microbiol. 23, 4778–4796. doi: 10.1111/1462-2920.15668, PMID: 34258839

[ref95] Zlatković S. Medić O. Predojević D. Nikolić I. Subakov-Simić G. Onjia A.. (2022). Spatio-temporal dynamics in physico-chemical properties, phytoplankton and bacterial diversity as an indication of the Bovan reservoir water quality. Water 14:391. doi: 10.3390/w14030391

[ref96] Zou S. Fu R. Deng H. Zhang Q. Gentekaki E. Gong J. (2021). Coupling between ribotypic and phenotypic traits of protists across life cycle stages and temperatures. Microbiol. Spectr. 9, e01738–e01721. doi: 10.1128/Spectrum.01738-21, PMID: 34817220 PMC8612162

[ref97] Zou K. Wang R. Xu S. Li Z. Liu L. Li M.. (2021). Changes in protist communities in drainages across the Pearl River Delta under anthropogenic influence. Water Res. 200:117294. doi: 10.1016/j.watres.2021.117294, PMID: 34102388

