# Supplementary material for: Spatiotemporal assembly and functional composition of planktonic microeukaryotic communities along productivity gradients in a subtropical lake
Source: Front Microbiol. 2024 Feb 19;15:1351772. doi: 10.3389/fmicb.2024.1351772 (PMC10909917; doi:10.3389/fmicb.2024.1351772)
Supplement: Supplementary file 2 [file Data_Sheet_2.docx]

*Supplementary materials 1*

**Spatiotemporal assembly and functional composition of planktonic microeukaryotic communities along productivity gradients in a subtropical lake**

Songbao Zou^1^, Qingping Lian^1^, Meng Ni^1^, Dan Zhou^1^, Mei Liu^1^, Xin Zhang^1^, Guangmei Chen^2^, Julin Yuan^1*^

^1^ *Key Laboratory of Healthy Freshwater Aquaculture, Ministry of Agriculture and Rural Affairs; Key Laboratory of Fish Health and Nutrition of Zhejiang Province; Huzhou Key Laboratory of Aquatic Product Quality Improvement and Processing Technology; Zhejiang Institute of Freshwater Fisheries, Huzhou 313001, Zhejiang, China*

^2^ *Zhejiang Fenghe Fishery Co. LTD, Qingtian County 323907, Zhejiang, China*

****Correspondence:***

*Julin Yuan, Freshwater Fishery Healthy Breeding Laboratory of Ministry of Agriculture, Zhejiang Institute of Freshwater Fisheries, 999 Hangchaoqiao Road, Huzhou, Zhejiang 313001, China.*

*Email:* *yuanjulin1982@163.com (J. Yuan)*

Running title: Freshwater microeukaryotic assembly and functional composition.

**
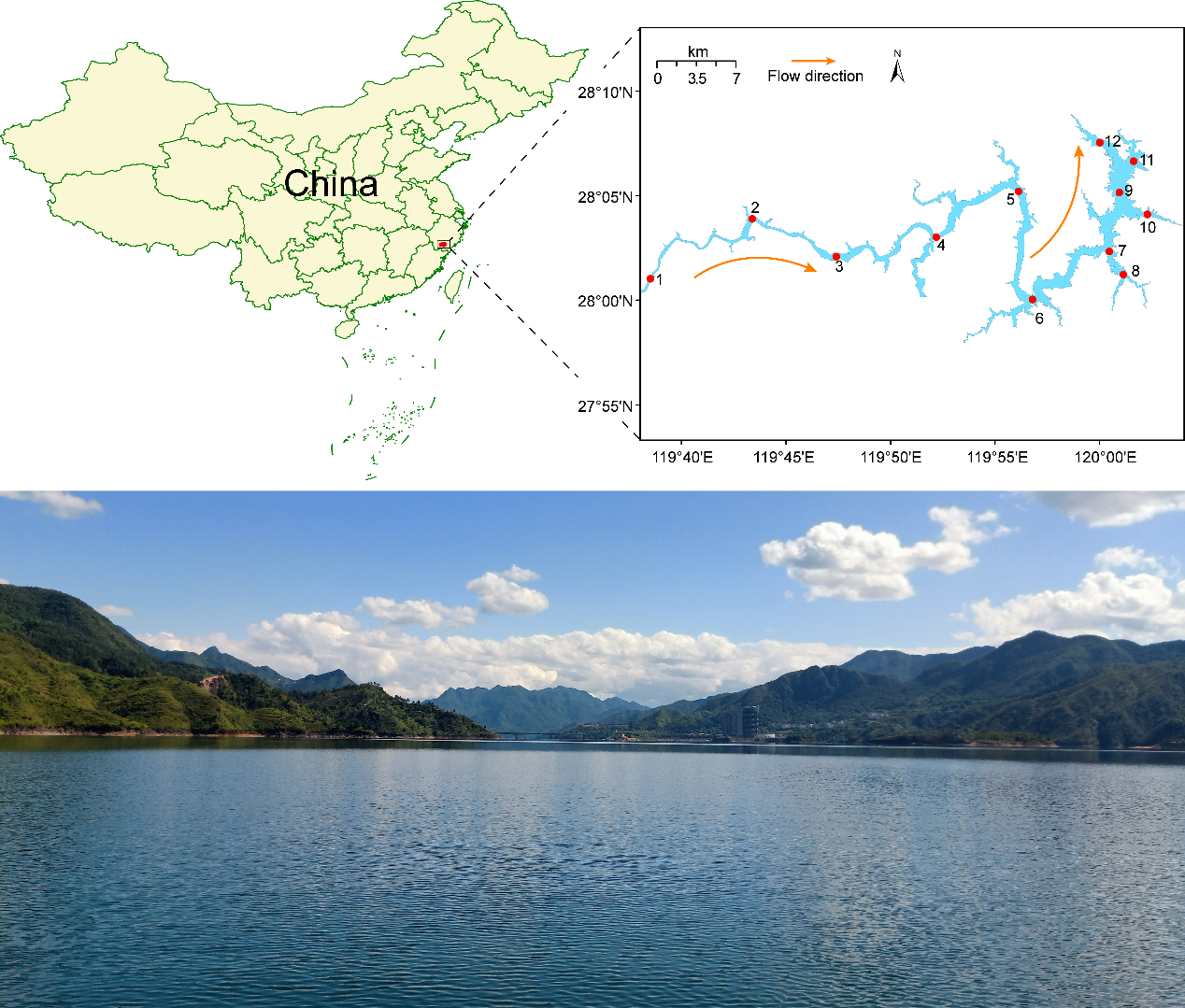
**

**Fig. S1** Map of sampling sites along the Qianxia Lake during both the spring and autumn using ArcGIS.


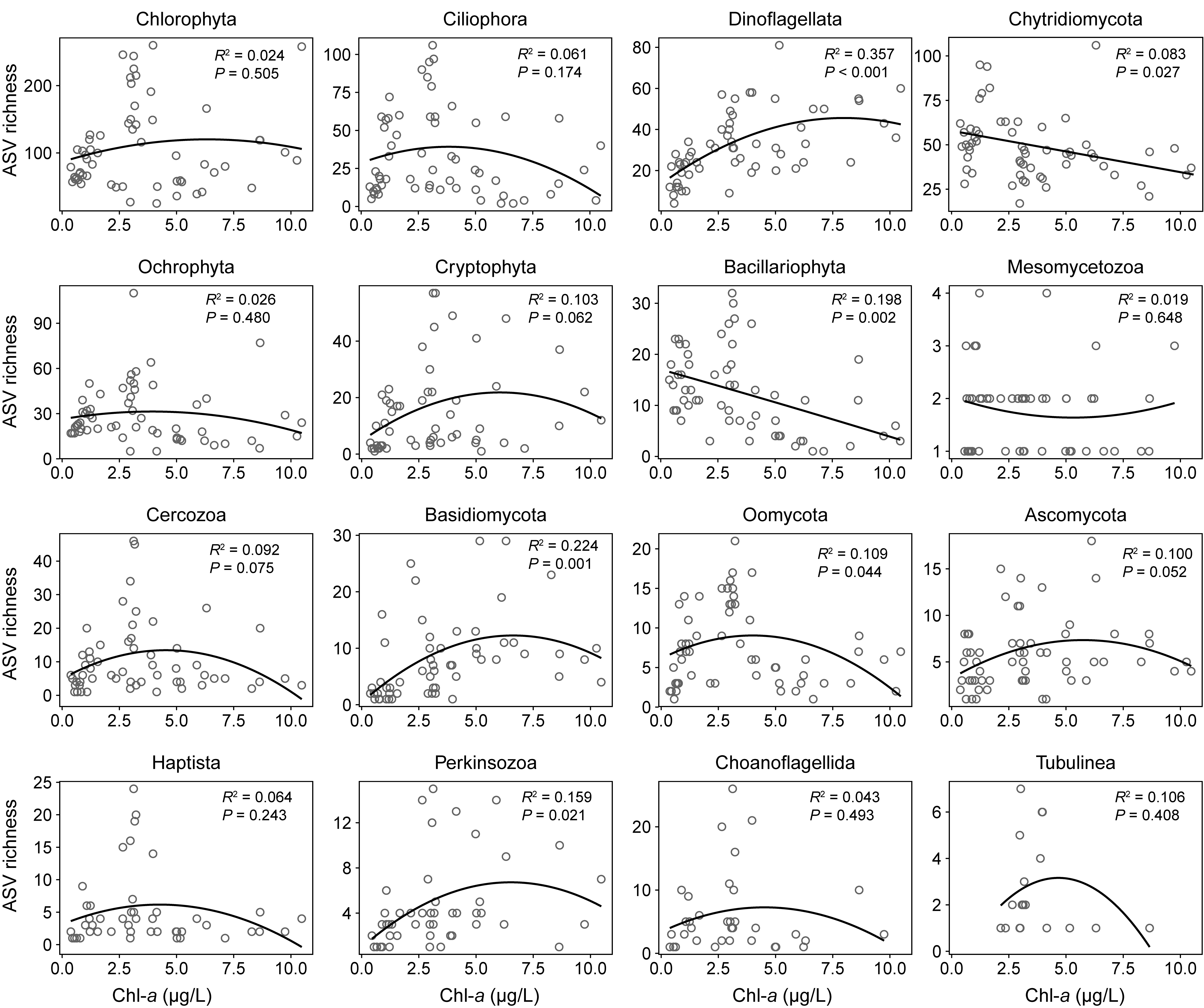


**Fig. S2** Relationships between ASV richness and chlorophyll-*a* concentration for dominant taxa of microeukaryotes. Notably, the Dinoflagellata, Basidiomycota, Oomycota, and Perkinsozoa exhibited significant hump-shaped patterns, while Chytridiomycota and Bacillariophyta showed significant linear relationship with chlorophyll-*a* concentration.


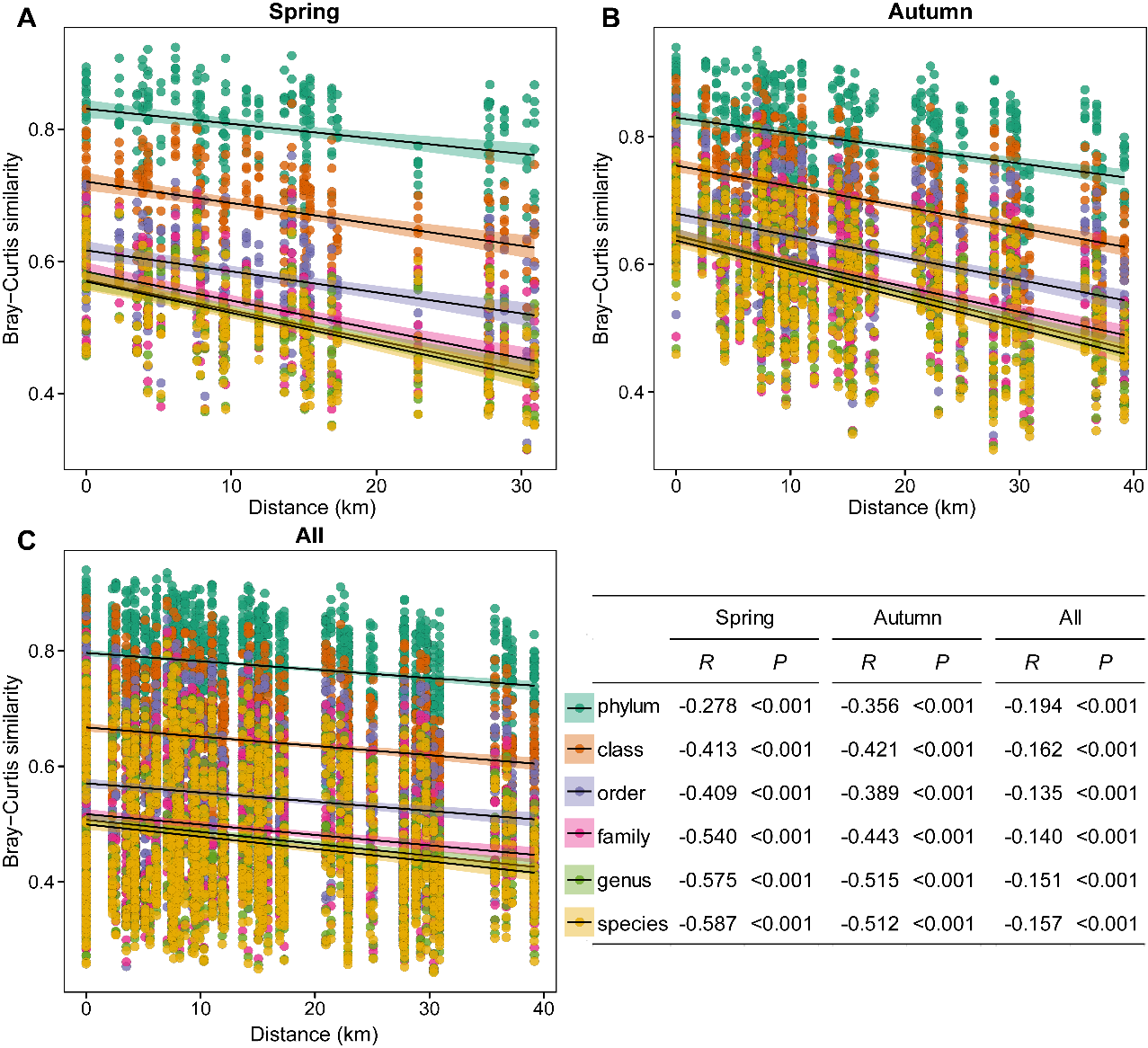


**Fig. S3** Distance-decay patterns of microeukaryotic community composition based on the Bray-Curtis similarity and geographical distance at different taxonomic ranks ranging from species to phylum levels in spring (**A**), autumn (**B**), and two seasons (**C**), respectively.


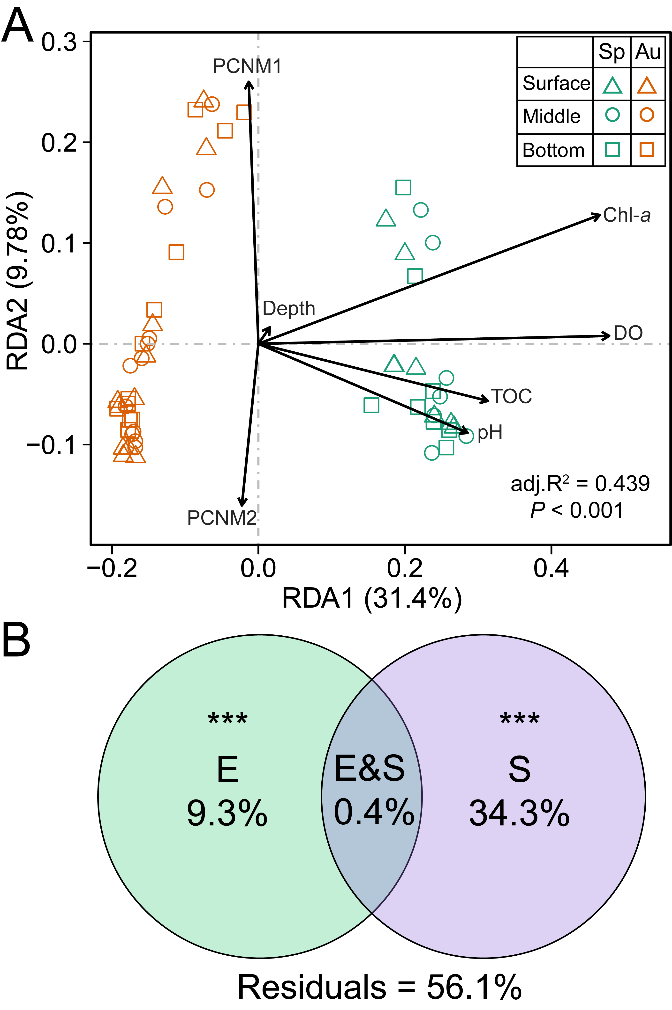


**Fig. S4** Redundancy analysis (RDA) and variation partitioning analysis (VPA) depict the effects of environmental and spatial (PCNM) factors on microeukaryotic communities variations for both seasons in Qianxia Lake. “E” represents relative contribution of environmental factors to community variation; “S” indicates relative contribution of spatial factors to community variation; “E&S” represents the shared explained variation; “Residuals” denote unexplained community variation. Statistical significance was assessed through permutational ANOVA (**P* < 0.05 and ****P* < 0.001).
